# Understanding the structural basis of HIV-1 restriction by the full length double-domain APOBEC3G

**DOI:** 10.1038/s41467-020-14377-y

**Published:** 2020-01-31

**Authors:** Hanjing Yang, Fumiaki Ito, Aaron D. Wolfe, Shuxing Li, Nazanin Mohammadzadeh, Robin P. Love, Maocai Yan, Brett Zirkle, Amit Gaba, Linda Chelico, Xiaojiang S. Chen

**Affiliations:** 1 Molecular and Computational Biology, Departments of Biological Sciences, Chemistry, University of Southern California, Los Angeles, CA 90089 USA; 2 Genetic, Molecular and Cellular Biology Program, Keck School of Medicine, University of Southern California, Los Angeles, CA 90033 USA; 3 Norris Comprehensive Cancer Center, University of Southern California, Los Angeles, CA 90033 USA; 4 Center of Excellence in NanoBiophysics, University of Southern California, Los Angeles, CA 90089 USA; 5 Department of Biochemistry, Microbiology, and Immunology, University of Saskatchewan, Saskatoon, SK Canada; 6 School of Pharmacy, Jining Medical University, 276800 Rizhao, Shandong China

**Keywords:** Biochemistry, Molecular biology, Structural biology

## Abstract

APOBEC3G, a member of the double-domain cytidine deaminase (CD) APOBEC, binds RNA to package into virions and restrict HIV-1 through deamination-dependent or deamination-independent inhibition. Mainly due to lack of a full-length double-domain APOBEC structure, it is unknown how CD1/CD2 domains connect and how dimerization/multimerization is linked to RNA binding and virion packaging for HIV-1 restriction. We report rhesus macaque A3G structures that show different inter-domain packing through a short linker and refolding of CD2. The A3G dimer structure has a hydrophobic dimer-interface matching with that of the previously reported CD1 structure. A3G dimerization generates a surface with intensified positive electrostatic potentials (PEP) for RNA binding and dimer stabilization. Unexpectedly, mutating the PEP surface and the hydrophobic interface of A3G does not abolish virion packaging and HIV-1 restriction. The data support a model in which only one RNA-binding mode is critical for virion packaging and restriction of HIV-1 by A3G.

## Abstract (web summary)

APOBEC3G (A3G) belongs to the DNA/RNA cytosine deaminase family that plays important roles in innate immunity against HIV and internal retroelements. Here the authors report the structures of two full-length A3G variants that provides insight into domain organization, multimerization, RNA binding, and viral restriction.

## Introduction

The AID/APOBEC proteins are a family of single stranded (ss) DNA/RNA cytidine deaminases that play important roles in diverse biological processes, including innate immune responses and cancer (reviewed in refs. ^1–3^ and references therein). APOBEC3s (A3s) are an APOBEC subfamily in mammals that restrict viruses, including retroviruses, endogenous retroelements^4–6^, Hepatitis B virus^7^, and Epstein-Barr virus^8^. The A3 subfamily in primates (including human) consists of seven members, three of which contain a single cytidine deaminase domain (CD) (A3A, A3C, A3H), and four of which (A3B, A3D, A3F, A3G) have double CD domains (CD1, CD2). The double-domain A3G, A3F, A3D, and single-domain A3H (haplotypes II, V, and VII) are active in restricting HIV-1 through deaminase-dependent and independent modes, with A3G being most potent among them^4,9–15^. For the double-domain A3s, the deaminase activity resides only on CD2, but the catalytically inactive CD1 is important for regulating nucleic acid binding, processivity, oligomerization, packaging into HIV virion, and effective anti-HIV activity^16–19^.

Despite the strong anti-HIV-1 activities of A3G, HIV-1 can induce A3G degradation through its viral infectivity factor (Vif) to establish infection (reviewed in refs. ^4,5^ and references therein). In the absence of HIV-1 Vif, A3G is packaged into budding virions, which not only induces mutations of the reverse-transcribed viral cDNA during infection^1,9,20,21^, but also inhibits the viral reverse transcriptase (RT) by binding to RT directly as well as binding to HIV RNA genome as a deamination-independent anti-HIV mechanism^11–14^. The N-terminal CD1, despite possessing no catalytic activity, is essential for virion packaging through an RNA binding-dependent mechanism^12,16,17,22^. However, the relationship between RNA binding and oligomerization is not clearly defined. The CD1 is also important for ssDNA substrate binding and can greatly enhance the deamination efficiency and processivity of full-length A3G^23,24^.

Vif binding to A3G directs the cellular Cullin 5 E3-ubiquitin ligase to ubiquitinate A3G, leading to proteasomal degradation^20,25–27^. Although the detailed molecular interactions between A3G and Vif remain to be determined, primarily only residues on CD1 have been shown to interact with HIV-1 Vif, particularly via the characteristic DPD^28–32^, among which a single residue at position 128 (D in human and K in monkey) governs the species-specific sensitivity of A3G to Vif within primates^22,28,33,34^. A K128D mutation on A3G from African green monkey or rhesus macaque (*Macaca mulatta*, or rA3G) induced sensitivity to HIV-1 Vif-mediated degradation, which changed the monkey A3G from inhibitory to sensitive to HIV-1 infection at a level similar to human A3G (hA3G)^33,35^. Thus, this species-specific polymorphism at position 128 creates an effective barrier to HIV transmission across primate species^36^, while also providing evidence that the antiviral functions and mechanisms of Vif-interaction of A3G are highly conserved among primates.

Newly synthesized A3G in cells multimerizes into large RNA-bound high molecular mass complexes (HMM) within 30 min after its production^21^, and the ability of A3G to bind RNA and multimerize is important for restricting non-LTR retroelements and HIV-1 infection^18,23,37–42^. The dependence of A3G multimerization on RNA binding has clearly been demonstrated as treatment of cell lysates expressing A3G with RNase A reduced the HMM complexes of A3G to a low molecular mass species (LMM), presumably to monomer, dimer, and tetramer forms^21,43^. Mutations of certain residues, notably W127, have also been shown to reduce RNA binding and multimerization^15,40^. However, while the role of RNA-binding in forming HMM complexes is apparent, it is not clear whether RNA binding is also required for the initial A3G dimerization prior to HMM formation. In addition, it is not clear whether CD1 is the sole domain involved in RNA binding and multimerization^15,17,40^ or if CD2 also plays a role in these processes^44,45^.

In order to understand the structural basis of domain organization and molecular regulation of the complex biological functions of A3G, we determined the double-domain structure of rA3G. Two structural conformations obtained from full-length (fl) rA3G reveal how the CD1 and CD2 domains interact with each other through a linker that has alternating conformations. These structures and mutational studies provide insight into how fl rA3G dimerizes through a CD1-CD1 interface that is important for mediating dimer formation as well as for RNA binding to the dimer junction. These results have elucidated the detailed functional interplay and molecular mechanisms of oligomerization and RNA binding that enable virion encapsidation and HIV-1 restriction. This high-resolution structural information of the fl rA3G is expected to provide important insights into the biological functions of A3G and the other double-domain A3s.

## Results

### Overall structural features of the fl rA3G

**Fig. 1:**
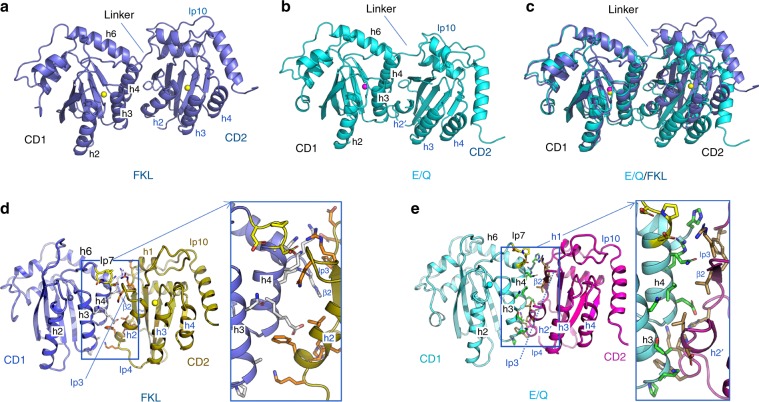
Overall structural features of the double domain A3G from rhesus macaque (rA3G). **a**, **b** Two different structures (FKL and E/Q) of the full-length rA3G (Supplementary Fig. 1 for 2nd structure assignment), showing that CD1 of both structures has a typical APOBEC fold with a Zn (sphere) at the active center. However, CD2 differs in the two structures. The CD2 of the FKL (A) has a canonical CD2 fold, but the CD2 of E/Q (B) re-folds helix 2 (h2) into a short 3_10_ helix and a dramatically altered Zn-center conformation with no Zn-coordinated. **c** Overlap of the two full-length rA3G structures based on CD1, which reveals that the CD2 domains in the two structures have different orientations relative to their CD1s. **d**, **e** The CD1-CD2 interface interactions of the FKL (D) and E/Q (E) structures. Insets show residues (in sticks) directly participating in CD1-CD2 domain interactions in the two structures respectively (see Supplementary Fig. 4 for list of interacting interface residues in FKL and E/Q structures).

A major hurdle in structural studies of fl double-domain APOBEC proteins has been poor solubility—wild-type forms are often purified either as non-homogeneous oligomers or large aggregates at higher concentration. In order to obtain well-behaved fl A3G for structural determination, we screened A3G homologs from different primate species and found that A3G from rhesus monkey (rA3G) had improved solubility. Two fl rA3G mutational constructs (termed FKL and E/Q) yielded well-behaved proteins and crystals in different conditions (Supplementary Table 1) that diffracted to 2.47 and 2.40 Å, respectively, (Supplementary Tables 1 and 2, Supplementary Fig. 1). Additional changes in these two constructs improved the solubility and yield of rA3G including replacement of an 8-residue CD1 loop 8 by the 4-residue loop 8 from hA3G-CD2 (as in both E/Q and FLK constructs, Supplementary Table 1, Supplementary Fig. 1), further mutations F126Y on CD1 loop 7, K180S/L184S on CD1 h6, and 8-residue deletion of the CD2 loop 3 (as in the FKL construct, Supplementary Table 1, Supplementary Fig. 1B). It should be noted that K128 of rA3G that acts as the barrier for cross-species transmission is mutated to an aspartic acid residue (D128) as in human A3G (hA3G)^36^. The constructs also contain catalytic E259 to Q/A mutation to avoid potential toxicity during recombinant protein expression. The mutated residues and their locations on the structure are shown in Supplementary Fig. 1B. In both structures CD1 and CD2 are folded into stand-alone domains connected by a short 5-residue linker (residues R194 to D198) (Fig. 1a, b). The two structures, despite overall structural similarity (Fig. 1a, b), show obvious differences in the CD2 conformations and the packing orientation between CD1 and CD2 (Fig. 1c, Supplementary Fig. 2A).

Both rA3G FKL and E/Q structures have the same canonical CD1 structure and superimpose well over each other (Supplementary Fig. 2B, rmsd of 0.445 Å). The much larger conformational differences in each CD2 domain result in a superimposition rmsd of 0.862 Å. When aligning the FKL and E/Q structures via their CD1 domain, the CD2 domain of the E/Q structure shows a rotation of ~29° compared with the CD2 of FKL structure, causing the E/Q CD2 domain to shift ~15 Å downward and ~8 Å toward CD1, and resulting in a much closer packing interaction with CD1 (Supplementary Figure 2 A). The overall CD1-CD2 packing interface has a buried surface area of ~623 Å^2^ for the FKL structure and ~700 Å^2^ for the E/Q structure. The tighter CD1-CD2 packing in the E/Q structure only leads to a slightly greater buried surface area than the FKL structure, likely because of the refolding and the missing density of the interface locating structural elements (h2-loop3) of its CD2.

### Two types of CD1 and CD2 domain interactions

While the FKL structure shows a canonical fold of its CD2 domain (Supplementary Fig. 2C), the E/Q structure shows significant alteration in CD2 conformation (Supplementary Fig. 2D), with major changes on helix 2 (h2), loop 3, loop 4, and the Zn-active center. The long h2 of the CD2 in the FKL structure (Supplementary Fig. 2C) has become a short 3_10_ helix (h2′) in the E/Q structure (Supplementary Fig. 2D), resulting in a disordered 14-residue stretch spanning parts of loop 3 and h2 (residues A246 to E259) (Supplementary Fig. 1A). The shift to a short 3_10_ h2 and extended random coil structure is necessary to avoid clashing in this tight packing conformation (Fig. 1e). However, this also disrupts the conformation of the CD2 Zn-active center within the regions of h2 and loop 3 that become disordered, resulting in no Zn-coordination (Supplementary Fig. 2D). The absence of Zn-coordination is observed in only one other APOBEC, the APOBEC3F (A3F) CD2 structure^46,47^. However, the Zn containing or absent A3F-CD2 structures are essentially identical, which also align well to several other APOBEC structures (Supplementary Fig. 2E), whereas the E/Q CD2 is unique in its refolded h2-loops 3 and 4 and the Zn-center conformation (Supplementary Fig. 2E, F).

While it is possible that the loss of Zn and the refolding of CD2 in the E/Q structure could be the result of the mutations in this construct, we investigated if the E/Q variant has defective catalysis activity. Reversion of E259Q in the E/Q construct back to the WT catalytic E259 (E/Q* construct) returned the variant full activity in deamination assay using HEK293T cell expression lysates (Supplementary Fig. 3). Additionally, E/Q* carrying individual mutations F126Y, K180S/L184S, or CD2Δloop3 (as in FKL structure) are all active, even though the activity of the FKL* construct (with E259A reverted back to E259) with the combined mutations is less than E/Q* and WT. These results suggest that even though the crystallized E/Q structure, lacking Zn in the CD2 domain, represents a catalytically inactive A3G structural state, reverting the catalytic residue back to E259 can fully restore its deaminase activity comparable to WT. Combined mutations in FKL partially affect its catalytic activity.

Due to the ~29° difference in the relative angle of rotation in packing between each domain in the FKL and E/Q structures, the detailed molecular interactions between CD1 and CD2 differ between the two structures. The CD1 and CD2 domains in the FKL structure interact with each other mainly through h3, h4, and loop 7 of CD1 and h1, h2, β2, and loop 3 of CD2 (Fig. 1a, d). In the E/Q structure, the two domains interact mainly through h3, h4, and h6 of CD1 with h2′, β2, loop 4 and loop 10 of CD2 (Fig. 1b, e). As a result, about 40% of the interacting residues between CD1 and CD2 differ in the two structures (see a complete list of the interacting residues in Supplementary Fig. 4), and even when the same residues are participating in this interaction, they often make different bonding contacts due to the change in packing angle between the two domains. The ability to pack the CD1 and CD2 domains with different angles and residue interactions indicates a certain degree of plasticity in the domain arrangement of fl A3G.

### Full length rA3G dimer and features of the dimerization area

**Fig. 2:**
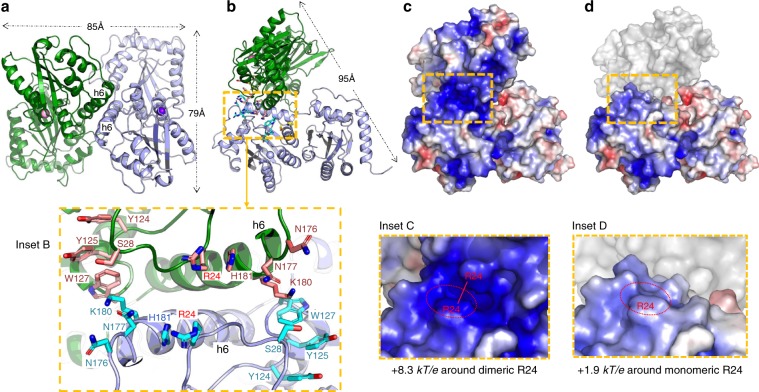
The overall features of a full-length rA3G dimer and the enhanced electrostatic potential at the dimer junction. **a**, **b** Two views of the dimeric rA3G of the E/Q structure, with the two subunits colored in green and light-blue. The dimensions of the dimer are indicated. Inset B a close-up view around the box area in yellow dashed line in **b**, showing 18 charged/polar and hydrophobic residues from two subunits circling the dimeric junction. Centered around the two R24, the location of these residues is suitable for binding single-stranded nucleic acids through charge interactions with phosphate backbone and hydrophobic stacking with the bases. **c**, **d** The surface charge calculated from rA3G dimer as viewed in **b** (**c**) or from the light-blue rA3G monomer in **b** (**d**). The averaged electrostatic potentials (EP) calculated for the rA3G dimer is + 8.3 kT/e for area centered around the R24 across the dimer junction (in dashed box in **c**, and Inset **c** in close-up), whereas the averaged EP for an rA3G monomer is 1.9 kT/e around the same R24 area (in dashed box in **d**, and Inset **d** in close-up) (see Methods on electrostatic potentials calculation, and calculated EP values are provided as a Source Data file), which indicates a significant enhancement of the positive EP around R24 area due to dimerization.

The E/Q construct was crystallized in different pH and salt conditions (Supplementary Table 1) but consistently showed the same structure and dimerization via CD1-CD1 interactions (Fig. 2a, b), primarily through direct packing of several residues on h6 (K180, L184, A187), loop 1 (I26) and loop 7 (F126, W127) from both subunits (Supplementary Fig. 5A, B). Interestingly, these interactions are identical to those previously reported for the rA3G-CD1 domain alone^18^, despite inclusion of the K128D mutation that is critical for turning rA3G from HIV-1 Vif insensitive to sensitive for degradation. It is worth noting that such dimerization of fl rA3G leads only to a small increase of the largest dimension from 85 Å of a monomer to 95 Å of a dimer (Fig. 2a, b).

A detailed examination of the residues aligned on either side of the dimeric interface area reveals two salient features. First, a total of 18 positive/polar and hydrophobic residues are aligned around the rA3G dimer junction in a way suitable for interaction with single-stranded nucleic acids such as RNA (Inset B in Fig. 2). These 18 residues are organized into two sets, with the first set in clockwise starting from the top monomer (in green), R24, H181, N177, N176, K180, and continuing to the bottom monomer (in light-blue) W127, Y125, Y124, and S28, and then with the mirrored set of these same residues. Second, through dimerization, the R24 and nearby positive residues K180 and H181 of each monomer are positioned closely in space, with about 7 Å distance between the two R24 residues. This spatial arrangement significantly enhances the local electrostatic potentials (EP) from around +1.9 kT/e as a monomer to +8.3 kT/e as a dimer (Fig. 2c and Inset C, Fig. 2d and Inset D). The presence of well-aligned residues suitable for binding single-stranded nucleic acids as well as the enhanced positive EP (PEP) around the observed dimer junction strongly imply that this area may bind RNA as a dimer, and suggests disruption of this A3G dimerization interface may impact RNA binding through disrupting the enhanced PEP (Fig. 2c) to the low PEP as in a monomeric form (Fig. 2d).

### Dimer mutation effect on RNA association and multimerization

Our previous study on the CD1 domain alone suggested the dimer-interface residues FWKL (F126, W127, K180, L184) may be involved in both dimerization and RNA binding, either through direct interaction with RNA or indirectly by generating an RNA binding surface via dimerization^18^. Inspection of the fl rA3G dimer structure reveals that, while the residues FWKLA (F126, W127, K180, L184, A187) directly participate in dimerization interactions (Supplementary Fig. 5A), only W127 is accessible for pi-stacking or hydrogen bonding with a nucleic acid base, suggesting that it is W127 that has the critical dual roles in dimerization or/and RNA binding, which has also been suggested by previous mutational studies^15,18,40,48^.

Loop 7 of rA3G is located near the dimerization interface and contains a set of hydrophobic residues (Y124, Y125, and F126) that pack with and likely stabilize W127 (Supplementary Fig. 5B). To address whether dimerization is needed for RNA association and to also consider the possible dual role of W127, we designed a set of dimer-interface mutants of rA3G that left loop 7 unchanged (Supplementary Table 3, Supplementary Fig. 5A, B). As such, only buried interface residues outside loop 7 were mutated, generating the mutants rM10, rM11, and rM15 (Supplementary Table 3). As a control, we also included a mutant rM9 with changes on both loop 7 and h6 of CD1 (F126A-W127A-A187Y) that was expected to result in a similar phenotype as the previously reported FWKL mutant of the CD1 alone^18^; i.e. disruption of both dimerization and RNA association. A near wild-type rA3G (WT) with a solubility-enhancing loop switching on CD1 loop 8 and its corresponding catalytically inactive mutant (construct E/Q) were also included (Supplementary Table 3). The RNA association and oligomerization status of these mutants after recombinant expression in *E. coli* were examined.

**Fig. 3:**
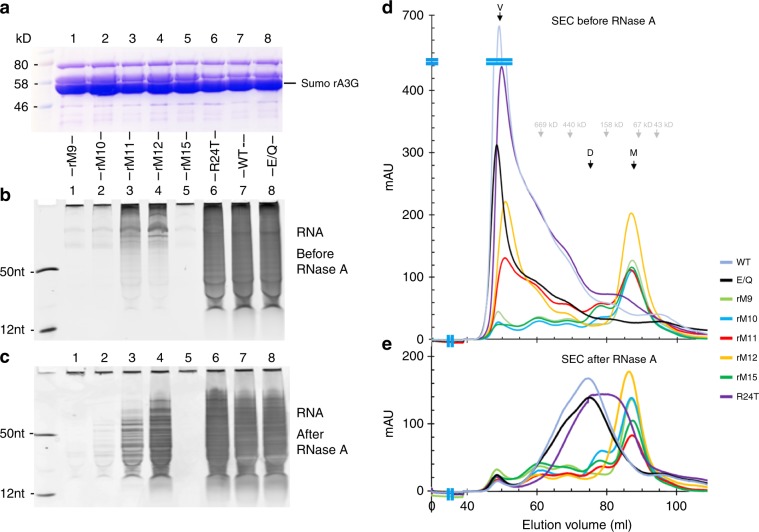
Probing dimerization and RNA association by targeted mutations of full-length rA3G. **a**–**c** The SDS-PAGE protein gel analysis of the His_6_-sumo-rA3G WT and various mutants after nickel affinity column purification (**a**), and 20% denaturing urea polyacrylamide gel analysis of RNAs associated with the proteins without RNase A treatment during purification (**b**) or with RNase A treatment during purification (**c**) (see methods for details). **d**, **e** Superdex-200 size exclusion chromatography (SEC) analysis of the sumo-rA3G WT and mutant proteins before (**d**) and after (**e**) RNase A treatment. The positions corresponding to void volume, dimer, and monomer are indicated with arrows. Source data for all panels are provided in the Source Data file.

After affinity column purification from the *E.coli* cell lysates, RNA association of sumo-rA3G fusion protein was analyzed by denaturing urea-PAGE (Fig. 3a–c). While the WT and E/Q mutant (lanes 7, 8 in Fig. 3b, c) had similar RNA association, all the dimer-interface mutants (rM10, rM11, rM15 in lanes 2, 3, 5, respectively, in Fig. 3b, c) had greatly reduced RNA association (lane 7) before or after RNase A treatment, with rM10 (T183D-L184D-A187Y), rM15 (I26A-K180S-L184S-A187E) and the control mutant rM9 showing little detectable RNA after going through the same purification process (lanes 1, 2, 5). Size exclusion chromatography (SEC) revealed that rM10 and rM15, as well as the control mutant rM9, eluted predominantly as a monomer before and after RNase A treatment (Fig. 3d, e; Supplementary Fig. 6A, B), confirming disruption of dimerization/multimerization. Thus, these results suggest that under the experimental conditions mutating residues buried within the interface not only disrupts dimerization, but also affects RNA association even when loop 7 is unchanged, likely due to the loss of the enhanced PEP as a result of dimer disruption (Fig. 2c, d).

Because we could not perform the same type of biochemistry assay to assess similar mutants in human A3G (hA3G) due to its poor solubility, we generated a rA3G-hA3G chimera mutant (h6 chimera) in which the rA3G CD1 h6 is replaced with hA3G CD1 h6 (see Supplementary Table 3). This h6 chimera behaved similarly as rA3G WT during purification in terms of dimer/multimerization before or after RNase A treatment (Supplementary Fig. 7A, B), as well as RNA association, especially after RNase A treatment (Supplementary Fig. 7C, D). It is worth noting that, as shown in Supplementary Fig. 7A, B, while H6 chimera protein also shifted to dimer (D) and monomer (M) fractions after RNase A treatment (SDS-PAGE gel in Supplementary Fig. 7D), its SEC profile shows more heterogeneous peaks than that of rA3G WT, possibly because the chimeric protein has three residues (Y181, I183, I187) from hA3G that are more hydrophobic than those in rA3G (H181, T183, A187), and thus less stable/soluble. These results suggest the possibility that CD1 h6 can be interchangeable between rA3G and hA3G, which leads to the presence of the same PEP area that has essentially the same set of surface residues on both proteins.

### PEP mutation effect on RNA association and multimerization

We then investigated if the enhanced PEP-surface formed around the dimer junction (Fig. 2. Inset b) is important for RNA association. Four positive/polar residues centered around R24 were mutated to generate rM12 (R24T-S28A-N176A-N177D, Supplementary Table 3). A mutant containing R24T alone was also included. The RNA association of rM12 was reduced compared to R24T and WT (lanes 4, 6, 7 in Fig. 3b, c), indicating a role of these residues within the PEP area in enhancing RNA binding. However, when compared to rM9 (F126A/W127A/A187Y), rM12 still had substantial amount of RNA association before and after RNase A treatment (lanes 1, 4 in Fig. 3b, c), possibly due to partial disruption of RNA binding to PEP area but not to other areas of rA3G. Unexpectedly, SEC analysis revealed that rM12 had a major monomeric peak even before RNase A treatment (Fig. 3d, e, Supplementary Fig. 6A, B), indicating disruption of dimerization/multimerization without directly mutating the dimerization interface. This negative effect of PEP mutation on RNA association and dimerization/multimerization was further confirmed by two more rA3G mutants containing PEP mutations, rM13 and rM14 (Supplementary Table 3, Supplementary Fig. 7). These two mutants showed different levels of disruption of RNA association (Supplementary Fig. 7A) and dimerization/multimerization even before RNase A treatment under the tested condition (SEC profiles and gels in Supplementary Fig. 7C).

Interestingly, even though R24T single mutant and WT showed similar amounts of associated RNA detected by RNA gels in Fig. 3b, c, the detailed SDS-PAGE analysis of their SEC peak fractions before RNase A treatment revealed that most of R24T protein distributed into the fractions spread around the monomeric peak (M) location (Supplementary Fig. 6A), which is in contrary to the WT that is mostly distributed in the void volume (V) fractions. These results indicate change of RNA association and multimerization behavior with a single R24T mutation within the PEP area, which is consistent with prior studies of R24A mutations^14,30,38,40^. Taken together, these results demonstrate that, under these assay conditions, residues within the enhanced PEP area of the dimer junction (R24/S28/N176/N177) play an important role in mediating RNA association, and RNA association through these residues is conversely required for stabilizing dimerization and subsequent higher order multimerization.

### Effect of PEP/dimer mutations on in vitro RNA/DNA binding

The fl rA3G structures reveal additional areas with positively charged residues outside the enhanced PEP area as shown in Fig. 2c. While these positively charged residues outside the PEP area may not play a major role in forming stable A3G-RNA complexes as observed from *E. coli* cell lysates, they may still contribute to binding the defined ssRNA and ssDNA substrates. To test this, each purified protein sample was subjected to extensive RNase treatment in order to remove as much bound RNA as possible, and the binding affinity towards 50 nt ssRNA or ssDNA oligomers was tested using a gel shift assay. All mutants displayed binding to 50 nt ssRNA or ssDNA, and the estimated dissociation constants (Kd, Supplementary Table 3) indicated that most mutants had reduced binding when compared to WT and E/Q. These results indicate that in this reconstituted system where high concentrations of proteins and nucleic acids were present, these various rA3G dimer-interface and PEP area mutants are still capable of binding to RNA and ssDNA through other residues outside the PEP area at the dimer junction.

Overall, the reduction of binding of these mutants is more severe for ssDNA binding than RNA binding. Interestingly, the deaminase assay using the purified proteins of these rA3G mutants (rM9-rM12 and rM15) revealed that these dimer-interface mutants and RNA-binding mutants all showed reduced deaminase activity compared with WT (Supplementary Figure 8). The lowered ssDNA binding of these mutants could account, at least partially, for the various levels of reduction of deaminase activity. However, the degree of lowered ssDNA binding does not appear to have a strict correlation with the disruption level of deaminase activity. For example, rM12 displayed the lowest ssDNA binding (Supplementary Table 3) but is not the one with the least deaminase activity (Supplementary Fig. 8).

### Effects of PEP/dimer-interface mutations on HIV restriction

The W127A mutation on human A3G (hA3G) is known to disrupt virion packaging and, hence, HIV restriction, likely by abolishing RNA binding mediated by this residue. However, studies that have induced packaging of hA3G W127 mutants through a Vpr peptide fusion have identified deficiencies in HIV restriction^15^. To gain further insights into the mechanism of HIV-1 restriction by hA3G, we designed additional hA3G mutants guided by the rA3G structure and mutational data. The intention of these hA3G mutations (Supplementary Table 4) was to disturb the intramolecular CD1-CD2 interactions (M2, M3, M4) (Supplementary Fig. 9A, B), disrupt the RNA binding around the dimer junction by mutating increasing numbers of polar/charged residues (M12, M13, M14), or to disrupt the protein-protein dimer interactions by mutating increasing numbers of buried residues (M6, M10, M11) (Supplementary Fig. 9C).

**Fig. 4:**
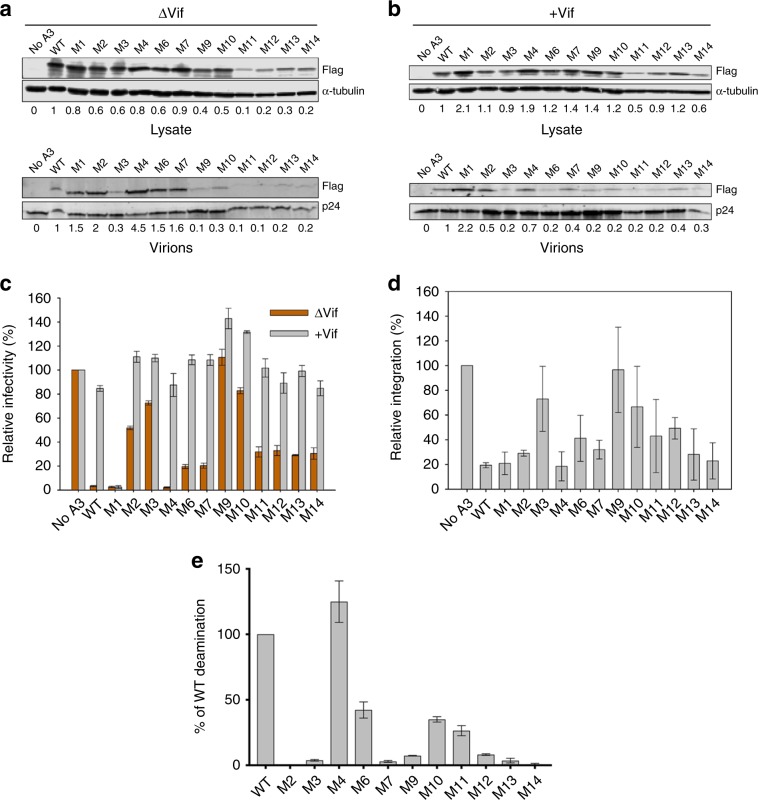
Virion encapsidation and restriction by A3G wild type and mutants. **a**, **b** Immunoblotting with FLAG antibody was used to detect transfected A3G wild type and mutants expressed in 293T virus producer cells and encapsidated into VSV-G pseudotyped virions in the absence, ΔVif (A) or presence, +Vif (B) of the A3 antagonist, Vif. The cell lysate and virion loading controls were α-tubulin and p24, respectively. The relative A3G levels shown below blots were calculated by setting the A3G wild type condition to 1 and determining the relative values of other lanes. One representative blot from three independent experiments is shown. **c** Infectivity in the absence or presence of Vif was measured by β-galactosidase activity in TZM-bl reporter cells. Results were normalized to the no A3 condition. Error bars represent the standard deviation of the mean calculated from three independent experiments. **d** The relative amount of proviral DNA integration in infected 293T cells in the presence of A3G wild type and mutants in comparison to the No A3 condition was determined by qPCR. Error bars represent the standard deviation of the mean calculated from at least two independent experiments. **e** The result of deaminase activity assay in lysates from 293T cells expressing hA3G and mutants. Error bars represent the standard deviations of the mean calculated from three independent experiments. Source data are provided in the Source Data file.

As expected, WT hA3G had no HIV-1 restriction activity in the presence of Vif, but fully restricted HIV-1 in the absence of Vif, and the Vif-resistant M1 (D128K) construct fully restricted HIV infection with or without Vif (Fig. 4a–c). On the other hand, The W127A containing mutant M9 (with mutations F126A/W127A/I187Y, Supplementary Fig. 9C) known to disrupt both RNA binding and dimerization had no restriction activity regardless of Vif presence (Fig. 4a–c). This is consistent with prior literature stating W127 is important for HIV restriction activity because of its RNA binding ability that is necessary for hA3G virion packaging and deamination-independent restriction of reverse transcription^14,15^. Indeed, even though its sensitivity to Vif-degradation appeared to be reduced (Supplementary Fig. 10A), ~5- to 10- fold less M9 mutant protein was packaged into virion in the presence or absence of Vif compared to WT hA3G (Fig. 4a, b). These results of WT and control mutants M1 and M9 of hA3G demonstrated full activity or lack of activity in our study.

For the mutants designed to affect the intramolecular CD1-CD2 interactions, multiple residues near or within the inter-domain interface on the CD2 side were mutated for M2 and M3 and on the CD1 side for M4 (Supplementary Fig. 9A, B). M2 and M3 both had significantly lower HIV-1 restriction activity in the absence of Vif (Fig. 4c). M2 carrying mutations on CD2 loops 1 and 3 around the interface with CD1 showed a complete loss of catalytic activity (Fig. 4e), likely because some of these mutated residues, such as H216, are important for ssDNA substrate interaction^49^. M3 carrying mutations on the CD2 interface with CD1, starting from the linker residues R194/H195 (Supplementary Fig. 9A, B), had only ~10% WT deaminase activity (Fig. 4e), possibly due to loss of proper CD2 interaction with CD1 to affect coordinated ssDNA substrate binding for efficient catalytic activity. M4 carrying multiple mutations around the inter-domain interface on CD1 displayed characteristics similar to WT indicating these mutations have no obvious effect on restriction ability. Interestingly, this mutation had full catalytic activity (Fig. 4e), suggesting certain plasticity between CD1-CD2 interface interactions for functions.

M12 and M13 were designed to disrupt only the RNA binding to the PEP area at the dimer junction, and M14 contains most of the mutations of M12 and M13 plus four additional R/K residues (K52/K63/R69/K76) to eliminate the remaining sole positively charged surface on A3G-CD1 (Supplementary Fig. 9C). Surprisingly, these mutants all showed ~70% HIV-1 restriction activity in the absence of Vif (Fig. 4c). Even though it was difficult to quantify Vif sensitivity due to persistent low expression level of M12, M13, and M14 in Vif-sensitivity assay (Supplementary Fig. 10A), all three mutants, similar to WT, displayed no HIV restriction activity in the presence of Vif (Fig. 4c), suggesting they were in fact still sufficiently sensitive to Vif-mediated degradation. The reason for only 70% HIV-1 restriction activity of M12-14 was not due to their lower steady state protein levels detected in HEK293T cell lysates (Fig. 4a, b), since transfection of less WT expression plasmid to achieve similar cellular expression levels as M12-M14 still resulted in ~4-fold more HIV-1 restriction from WT (Supplementary Fig. 10B). This is consistent with the deamination-independent restriction activity, as these three mutants had disrupted deamination activity (Fig. 4d, e). Especially M14 had a complete loss of deaminase activity (Fig. 4e). Consistent with this was the background mutation rate of M14 based on the proviral DNA sequencing results (Supplementary Table 5). And yet it displayed ~70% HIV-1 restriction activity. These results clearly demonstrate that the residues mutated in M12-14 to disrupt RNA binding in the PEP and nearby areas on CD1 showed only partial defect in restricting HIV, indicating they retained certain level of virion encapsidation and HIV-1 restriction, which is in contrast to the role of RNA binding mediated through mutations containing W127 in the M9 mutant that is critical for virion packaging and HIV restriction (Fig. 4a–c).

Those mutants intended to disrupt only the protein-protein dimer interactions with increasing number of mutations are from M6 to M11 (Supplementary Table 4, Supplementary Fig. 9C). M6 and M7 displayed only partial loss of restriction activity in the absence of Vif, despite of the 95% loss of catalytic activity for M7 due to the additional three mutations on CD2 (Fig. 4e). Therefore, the mutations in M6 and M7 are not critical for HIV-restriction. M10 showed more severe phenotype in HIV-1 restriction in the absence of Vif even though it retained some catalytic activity (Fig. 4e), suggesting mutations on M10 are important for virion packaging and HIV-restriction. M11 is similar to M10 in deaminase activity, but with less severe phenotype in terms of restriction activity and integration (Fig. 4c, d). It is possible that M11 retained more RNA binding ability than M10 (from rM10-11 of rA3G mutant analysis, Fig. 3a–e), resulting in less severe phenotype. Taken together, these results showed that mutating residues within the buried protein-protein dimer interface alone still retained HIV-restriction activity at reduced levels (Fig. 4c), and the disruption of deaminase activity of these mutants (Fig. 4e) may partially account for the reduction of HIV restriction activity. This again is in contrast to the M9 mutant that displayed no HIV restriction activity (Fig. 4a–c).

## Discussion

We report here the full-length structures of rA3G that represent the first double domain APOBEC structure. The structures reveal that CD1 and CD2 domains can form different interactions through a flexible linker to pack with each other at different angles. Two different CD1-CD2 packing orientations are observed in the two fl rA3G structures, suggesting the possibility for a certain degree of plasticity between the two domains, consistent with a prior computational simulation study^50^. M4 mutant carrying mutations on the CD1 interface with CD2 to alter the packing interactions still displayed full deaminase activity (Fig. 4e) and full HIV-1 restriction in the absence of Vif (Fig. 4c). Also revealed by the structure is a conformational switch (or plasticity) within the CD2 domain: almost all of h2 and most of loop 3 refold to accommodate the change in CD1-CD2 orientation. This plasticity likely enables diverse interactions with ssRNA and ssDNA, which may help explain the capability of A3G to bind various RNA species^51^, to be sensitive to allosteric inhibition of ssDNA binding^52^, to wrap or bend ssDNA^23,52^, and to undergo processive scanning on ssDNA^53^. Alternatively, the different CD1-CD2 packing angle could also be the result of mutational effects on the packing.

Full-length rA3G dimerizes through a hydrophobic CD1-CD1 protein interface and dimerization generates an area with intensified positive electrostatic potentials (PEP). Mutational and functional studies guided by the structure showed that the buried A3G hydrophobic dimer interface interactions mediated by CD1 h6 were required for RNA binding to form protein-RNA multimeric complexes and affected deaminase activity, but still retained certain levels of virion packaging and HIV restriction, suggesting a non-critical role for packaging and HIV restriction. Remarkably, dimerization dramatically enhances the PEP by positioning positively charged residues around R24 from two monomers in close proximity (Fig. 2c Inset c, Fig. 2d Inset d). This phenomenon is reminiscent of observations for the dsDNA minor groove, where the negative electrostatic potentials (NEP) can be enhanced by positioning the two phosphate-backbones closer^54^. The increased PEP after dimerization, together with the well-aligned residues suitable for binding nucleic acids around the dimer junction, suggests ssRNA binds to this region when dimerization occurs. Mutations of residues buried inside the dimer interface of rA3G (rM10, rM11, and rM15, Fig. 3) demonstrated that disrupting the hydrophobic dimerization interactions also disrupted RNA binding, providing further support for this hypothesis. Thus, these two functions appear to be intricately linked, and could possibly affect each through positive feedback effects, such that dimerization leads to increased PEP, leading to stronger RNA binding, which further stabilizes the dimerization. This is different from the A3H dimer structure, in which dsRNA is present between two protein molecules with no direct protein-protein contacts^55–57^.

Conversely, mutations of residues on the PEP surface around the dimer junction but away from the buried hydrophobic dimer interface showed disruption of not only RNA binding, but also dimer formation (rM12 in Fig. 3, Supplementary Table 3), demonstrating that RNA binding to the dimer junction area through the enhanced positive EP is critical for a stable dimer formation. Mutation of R24 alone on the PEP area caused relatively minor effect on RNA-associated oligomerization (compare R24T with WT in the SEC profiles and gels of Supplementary Fig. 6A), which is consistent with previous finding that R24A mutation alone did not cause major disruption of RNA binding or multimerization^14,30,38,40^. Thus, multiple mutations within the enhanced PEP area may be needed to produce major disruption of RNA binding to PEP. Areas outside the PEP area, including the positive patch centered around h2 of CD1 (patch-h2, Supplementary Fig. 9C), can also bind RNA as native gel shift assay showed that purified PEP mutant proteins still bind 50 nt RNA at high protein concentrations (Supplementary Table 3). However, it appears this RNA binding outside the dimer junction PEP is not involved in stable dimer formation of A3G. Thus, our data provides direct evidence for a role of a subtype of RNA binding in A3G dimerization/multimerization.

Based on the high-sequence homology between rA3G and hA3G, the rA3G structure is used as a surrogate to guide the structure/function study of hA3G relating to HIV-restriction in HEK293T cells. Similar mutations of the dimer-interface or the PEP surface as in rA3G were made in hA3G (Supplementary Table 4) and their HIV restriction activity were examined. Surprisingly, mutations of hA3G within the buried hydrophobic dimer-interface (M6, M10, M11, Supplementary Table 4) or the PEP surface at the dimer junction area (M12, M13) all displayed some level of HIV-1 restriction in the absence of Vif (Fig. 4c). Combining the mutations on the PEP area with four additional K/R residues on the remaining positive patch of CD1 (M14; see patch-h2, Supplementary Fig. 9C) also displayed ~70% HIV restriction activity. The fact that these mutants display partial HIV restriction activity indicates that they still retain certain level of virion encapsidation. In contrast, M9 mutant (F126A-W127A-I187Y) (Supplementary Table 4) displayed no HIV restriction activity and no virion encapsidation, likely due to the F126A/W127A mutations. This is consistent with earlier reports^15,40,48^ showing that W127 is critical for both RNA binding and dimerization, as well as for HIV restriction. Taken together, RNA binding to A3G can be mediated through different areas and residues, each of which may have distinct roles, such as the RNA binding to PEP area for stable dimer formation but not for virion encapsidation, the RNA binding to W127 on loop 7 of CD1 for virion packaging, and the RNA binding to the positive patch-h2 of CD1 for other functions yet to be identified.

Despite the importance of W127 at the dimerization interface in mediating virion encapsidation, these results from the mutations centered around PEP and dimer-interface suggest that dimerization per se and the type of RNA binding mediated through the PEP area and the R/K-rich patch h2 are not critically required for the proposed deamination-independent restriction mechanisms that involve RNA binding and inhibition of reverse transcription^11–15^. These are likely instead performed by other RNA binding modes, such as RNA interactions mediated by W127, or direct RT binding through CD1 protein surfaces outside those mutated here^14^.

Further investigation into Vif sensitivity and deamination activity of these hA3G mutants showed that partial HIV restriction activity in the absence of Vif occurs despite the decreased or abolished deamination activity of the mutants. Surprisingly, mutations within the buried dimer-interface or on the enhanced PEP surface all showed significant loss of deaminase activity compared to WT, both through deamination by cell lysate assays from HEK293T cells expressing these mutant proteins and direct sequencing of integrated proviral DNA (Supplementary Table 5), with M14 notably having no detectible deaminase activity despite HIV-1 restriction activity. This effect of lowering the deaminase activity by mutating the dimer interface or the enhanced PEP area is also demonstrated by the in vitro deaminase assay using purified proteins from the equivalent rA3G mutants (Supplementary Fig. 8). These results provide further evidence of the importance of deaminase-independent HIV restriction activity.

While we showed that PEP and dimer-interface mutations of hA3G may impact HIV restriction activity mainly through affecting the deaminase activity and retain HIV restriction in deaminase-independent manner, dimerization and RNA-binding-dependent multimerization of A3G is shown to be critical for internal retroelement inhibition (SINEs and LINEs) by several groups^6,37,38^. Therefore, the biological relevance of dimerization (and subsequent multimerization) may be critical for restricting internal retroelements than for external HIV-1.

In summary, the studies of fl rA3G reported here have provided structural basis for the long-standing enigmatic questions regarding the dimerization and multimerization process, interdependence between dimerization and RNA binding, and the intricate roles of different modes of RNA binding for dimerization, deaminase activity, virion encapsidation and HIV restriction. These results will provide help for the future understanding of the important biological functions of A3G and other double-domain APOBECs in anti-viral and cancer mutation, and future design of anti-HIV/AIDS and anti-cancer therapeutics.

## Methods

### Protein expression and purification

The gene encoding rA3G of rhesus monkey (Macaca mulatta, accession AGE34493) was codon optimized and cloned into pET SUMO vector (ThermoFisher) to generate a sumo fusion that carries an N-terminal 6xHis tag and a PreScission protease cleavage site. Mutations of rA3G were introduced by a PCR-based method using TaKaRa PrimeSTAR mutagenesis basal kit (Takara). rA3G expressing *E.coli* Rosetta™(DE3)pLysS cells were cultured at 37 °C in LB medium supplemented with 50 μg/ml kanamycin until OD_600_ reached 0.3, the growth temperature was then lowered to 16 °C and *E. coli* cells were further cultured. Protein expression was induced by addition of 0.1 mM isopropyl β-D-thiogalactopyranoside when OD_600_ reached 0.7-0.9. *E. coli* cells were further cultured at 16 °C overnight and harvested by centrifugation. Cell pellet was resuspended in buffer A (25 mM HEPES, pH 7.5, 500 mM NaCl, 20 mM MgCl_2_, and 0.5 mM TCEP), lysed by sonication or French Press in the presence of RNase A (60 μg/ml), and then centrifuged at 29,000 *× g* for 1 h. 6xHis sumo fusion was captured by Ni-NTA agarose gravity-flow chromatography followed by a series of wash containing 25 mM imidazole in buffer A, 50 mM imidazole in buffer B (50 mM HEPES, pH 7.5, 500 mM NaCl, and 0.5 mM TCEP), 1 M NaCl in buffer B, and two times 50 mM imidazole in buffer B. Sumo rA3G was then eluted with 0.5 M imidazole in buffer B containing 250 mM NaCl, concentrated, and switched to buffer B containing 1 mM EDTA. Five microliter RNase A (100 mg/ml) was added to the concentrated Ni elution fraction and left in cold room overnight followed by HiLoad™ 16/60 prep column on Superdex 200 gel filtration chromatography (GE Healthcare) in buffer B. The peak fractions of sumo rA3G were concentrated, mixed with PreScission protease to cleave the His_6_-SUMO-tag, and further purified by HiLoad™ 16/60 Superdex 75 to obtain the cleaved FKL and E/Q. They were further concentrated to 11 mg/ml for the cleaved FKL and 15 mg/ml for the cleaved E/Q and stored at −80 °C until use. The cleaved E/Q was also purified using Tris-HCl at pH 8 to replace HEPES.

### Crystallization and structure determination

Sitting-drop crystallization screening plates were set at 18 °C using ARI Crystal Gryphon Robot (ARI) and crystallization screening solutions (Qiagen and Hampton Research). The purified FKL was crystallized in a range of conditions. The crystallization conditions were further optimized and the diffraction-quality crystals were obtained under the condition of 0.1 M MES, pH 6.9 and 8% PEG 20 K using the sitting-drop vapor diffusion method at 18 °C. The purified E/Q was also crystallized in several conditions and it was further optimized using the hanging-drop vapor diffusion method at 18 °C. The diffraction-quality crystals were obtained under several conditions: (1) 50 mM MES, pH 5.2, 1.7 M lithium sulfate and 10 mM magnesium chloride with protein to precipitant ratio of 1:1, (2) 50 mM HEPES, pH 7.0, 1.6 M ammonium sulfate and 10 mM magnesium chloride with protein to precipitant ratio of 1:1, and (3) Tris pH 7.4, 1.9 M ammonium sulfate, and 25 mM magnesium chloride with protein to precipitant ratio of 2:1. The third condition was used when HEPES buffer was replaced by Tris-HCl pH 8 during protein purification. Glycerol (24%–30%) was used as cryoprotectant and all crystals were flash-frozen in liquid nitrogen and stored until data collection. Diffraction data were collected at beamline 23ID-D of GM/CA@APS. Data were processed and scaled using HKL2000. Initial phase information was obtained by molecular replacement method with PHENIX using the crystal structures of rA3G N-terminal domain PDB 5K81 [https://www.rcsb.org/structure/5k81] and hA3G catalytical domain PDB 3IR2 [https://www.rcsb.org/structure/3IR2] as search models. The structural model was refined using PHENIX and modified with COOT. Data collection statistics and refinement parameters are given in Supplementary Table 2. Structure figures were prepared with PyMOL molecular visualization system.

### Calculating electrostatic potentials at the dimer junction

PDB files for either the monomer or dimer full-length A3G were converted to PQR by the PDB2PQR standalone tool using the AMBER forcefield. APBS was used to calculate electrostatic surface potential using a grid centered on R24 residue located within the dimer interface; potentials were calculated using a non-linear Poisson-Boltzman model at 310 K and using 150 mM salt in the solvent^54^. Virtual atom probes were randomly placed near R24 within this interface but above the electrostatic surface and the resulting potentials were averaged to calculate the overall increase in positive potential upon dimerization in this local domain.

### FPLC analysis

Sumo rA3G constructs containing the desired mutations were prepared and purified by Ni-NTA agarose (QIAGEN) using the same protocols as described in the previous protein purification section but under the condition with or without RNase A treatment along the process. Oligomeric status of the Ni elution fraction was analyzed by SEC with HiLoad™ 16/60 Superdex 200 column in buffer B. Separation of protein species by SEC was visualized with 8% SDS-PAGE using samples from every fourth fractions covering elution volume 45 - 98 ml. HiLoad™ 16/60 Superdex 200 gel filtration column was calibrated in buffer B using defined protein standards (GE Healthcare).

### RNA gel of purified proteins plus/minus RNase A treatment

The concentration of the rA3G protein samples prepared above were normalized, mixed with 2× formamide loading buffer (95% formamide, 25 mM EDTA), and loaded on to 20% urea denaturing gel to separate RNA species. Gel was stained by SYBR™ Gold Nucleic Acid Gel Stain and the image was visualized by Typhoon RGB Biomolecular Imager (GE Healthcare).

### EMSA

It is known that the RNA bound to A3G affects RNA or ssDNA binding in EMSA study, so the protein samples were prepared with the aim of removing the bound RNA as much as possible by heavy RNase A treatment during protein purification as described in the first section. Briefly, RNase A (60 μg/ml) was included during cell lysis by sonication and also in the first three washing buffer (containing 10 μg/ml RNase A) in Ni-NTA agarose (QIAGEN) gravity-flow chromatography. Sumo rA3G was then eluted with 0.5 M imidazole in buffer B containing 250 mM NaCl, concentrated, and switched to buffer B containing 1 mM EDTA. Five μl diluted RNase solution containing 100 mg/ml RNase A and 100 U/μl RNase T1 was added to 0.5 ml concentrated Ni elution fraction and left in cold room overnight followed by HiLoad™ 16/60 Superdex 200 gel filtration chromatography (GE Healthcare) in buffer B. The peak fractions were combined and concentrated. Protein concentration was measure by NanoDrop and normalized. 6-FAM labeled oligonucleotide at 10 nM (50 nt ssRNA or 50 nt ssDNA)^58^ was titrated by sumo-rA3G in 10 μl reaction volume containing 50 mM HEPES pH 7.5, 250 mM NaCl, 1 mM DTT, 2.5 mM EDTA and 10% glycerol. The reaction mixture was incubated on ice for 10 min and analyzed by 8% native PAGE. Typhoon RGB Biomolecular Imager (GE Healthcare) was used to visualize the image and ImageQuant TL (GE Healthcare) was used for image quantification.

### A3G deamination assay

HEK293T cells were grown in DMEM media and plated in 12-well trays for transfection; upon reaching approximately 70% confluency cells were transfected with 0.5 μg of each A3G construct using X-TREME gene 9 transfection reagent (Sigma). Expression of A3G continued for 48 h before the cells were lysed using 100 µL of MPER-halt solution (Thermo) per well. Resulting lysates were normalized using a BCA quantification assay (Pierce) with adjustment made by adding additional MPER-halt buffer to the level of whichever had the lowest total protein concentration. The normalized samples were then analyzed and quantified by Western blot to confirm that the levels of A3G expression were similar. This process was repeated by adjusting the DNA concentration for the second round to account for large deviations in overall expression levels. After the second round of transfection and Western analysis, the lysates were normalized with empty vector transfection control lysate to the observed lowest level of expression and the samples were used for in vitro deamination. Cell lysates were allowed to react with DNA deamination mix (25 mM Hepes 7.5, 2 mM DTT, 0.1% Triton X-100, 0.1 mg/mL RNase A, 300 nM FAM-50-CCC single stranded DNA, and 50 mM NaCl) for a total of three repeats of each construct. After one hour, reactions were stopped by heating and UDG was allowed to incubate for one hour, followed by abasic hydrolysis by addition of NaOH. Samples were run on 20% urea denaturing PAGE and quantified by dividing the lower product band by the sum of the product and substrate bands.

### Vif-mediated degradation assay of A3G

Codon-optimized hA3G WT and mutants were cloned into pcDNA3.1(+) mammalian expression vector with FLAG-tag at N-terminus. Codon-optimized, untagged Vif from HIV-1 pNL4-3 was also cloned into pcDNA3.1(+) vector. Cloning and mutagenesis were performed with In-Fusion cloning HD Cloning Plus (Clontech) and PrimeSTAR MAX DNA Polymerase (Clontech) by following manufacturer’s instruction. The sequences of all the constructs were verified by DNA sequencing (Genewiz). For analysis of the effect of Vif on cellular A3G level, pcDNA-FLAG-hA3G mutants (100 ng) were co-transfected with either pcDNA-Vif or pcDNA3.1(+) empty vector (200 ng) into HEK293T cells (ATCC) in 12-well plates by using X-tremeGENE 9 DNA Transfection Reagent (Roche). At post-48 h transfection, the cells were washed once with PBS, and lysed in RIPA buffer (Sigma) with 1x complete protease inhibitors (Roche). The lysates were then subjected to Western blot with anti-FLAG M2 mAb from mouse (Cat # F3165, Sigma, 1:3,000), anti-α-tubulin mAb from mouse (Cat # GT114, GeneTex, 1:5,000) and anti-Vif mAb from mouse (NIH AIDS Reagent Program #319, 1:2,000) as primary antibodies. Cy3-labelled goat-anti-mouse mAb (Cat # PA43009, GE Healthcare, 1:3,000) was subsequently used as a secondary antibody to detect the signal. Cy3 signals were detected and visualized with Typhoon RGB Biomolecular Imager (GE Healthcare).

### Single-cycle replication assays

VSV-G pseudotyped HIV-1 NL4-3 ΔVif ΔEnv or HIV-1 NL4-3 ΔEnv were produced by transfecting 1 × 10^5^ HEK293T cells per well of a 12-well plate. The HEK293T cells were maintained in DMEM with 10% FBS and Penicillin/Streptomycin. GeneJuice (Novagen) transfection reagent was used as per manufacturer’s protocol. Cells were transfected with 500 ng of pHIV-1 NL4-3 ΔVif ΔEnv or HIV-1 NL4-3 ΔEnv, 180 ng of pMDG, which expresses VSV-G^59,60^, and between 60 and 250 ng of A3G expression plasmid to achieve similar expression levels of A3G wild type and mutants. The following pcDNA3 constructs were transfected for expression of FLAG-tagged A3G mutants: A3G-WT, A3GM1, A3GM2, A3GM3, A3GM4, A3GM6, A3GM7, A3GM9, A3GM10, A3GM11, A3GM12, A3GM13, and A3GM14. To equalize the amount of plasmid DNA transfected, empty pcDNA3 was used. After 24 h the media was changed and virus containing supernatants were harvested 24 h after the media change. Supernatants were filtered through a 0.45 μm polyvinylidene difluoride (PVDF) syringe filter. For infection of a reporter cell line to determine infectivity 1 × 10^4^ cells per well of a 96-well plate containing TZM-Bl cells were infected with virus normalized by p24 levels (QuickTiter Lentivirus Titer Kit, Cell Biolabs Inc.) in the presence of 8 μg/mL polybrene. Forty-eight hours after infection the cells were washed with PBS and infectivity was measured through colorimetric detection using a β-galactosidase assay reagent (Pierce) and spectrophotometer. Infectivity of each virus was compared by using the infectivity of the No A3 condition as 100%.

### Proviral DNA integration assay

Methods to quantify the integrated proviral DNA were adapted from previous publications^15,61,62^. For infections, 1 × 10^5^ HEK293T cells per well of a 12-well plate were infected by spinoculation (1 h at 800* × g*) and in the presence of polybrene (8 μg/mL) with HIV produced from the single-cycle replication assays. DNA was extracted after 24 h using DNazol according to manufacturer’s instructions. The DNA was then treated with DpnI and 50 ng was used in a PCR with primers qAlu1 and qAlu2 5 and Nef FWD-OUT (5′GGG TCA GAT ATC CAC TGA CCT TTG G) to ensure amplification of DNA from integrated HIV genomes. The PCR cycle used a Taq Master Mix (Qiagen) and an annealing temperature of 50 °C and extension time of 3 min. The PCR was then diluted 40-fold and used as the template in a qPCR with 9 pmol/mL of each primer (Primer 1, 5′GTA CCA GTT GAG CCA GAT AAG G; Primer 2, 5′GCT GTC AA ACCT CCA CTC TAA C) and 0.25 pmol/mL of the probe (5′FAM-TGT TAC ACC -ZEN- CTG TGA AGC CTG CAT-IABkFQ). Reactions were performed in triplicate with TaqMan Gene Expression master mix (Applied Biosystems). Cycling conditions were 10 min at 95 °C, followed by 40 cycles of 15 s at 95 °C and 1 min at 60 °C using a Bio-Rad CFX-96. The copy numbers in each sample were normalized for DNA input using human RNase P (Applied Biosystems).

### Quantitative immunoblotting

HEK293T cells expressing No A3 or FLAG-tagged A3G mutants from the single-cycle infectivity assays were detected using rabbit anti-FLAG (1:1000, Cat # H6908, Sigma). A3s were detected in cell lysates and virions. Cells were lysed using 2x Laemmli Buffer and 30 μg total protein was used. Virus was concentrated using Retro-X (Clontech, Cat # 631456) following the manufacturer’s protocol and 12.5 μL of concentrated virus was used. Loading controls for cell lysates (α-tubulin, 1:1000, mouse monoclonal cat #T-8203, Sigma or rabbit polyclonal Cat # PA1-20988, Invitrogen) and virus (p24, Cat #3537, NIH AIDS Reagent Program) were detected with mouse monoclonal antibodies. Secondary detection was performed using Licor IRDye antibodies produced in goat (IRDye 680-labeled anti-rabbit 1: 10000 Cat # 926-68071 and IRDye 800-labeled anti mouse 1:10000 Cat # 926-32210). Immunoblots were quantitatively analyzed where indicated in figures. Quantitation of band intensities was performed using Image Studio Software with normalization of each experimental lane to its respective α-tubulin or p24, which was detected in parallel on the same blot. Relative expression levels were then determined by comparison to a control sample set at 1.

### Reporting summary

Further information on research design is available in the Nature Research Reporting Summary linked to this article.

## Supplementary information

Supplementary information

Peer Review

Reporting Summary

## Data Availability

The data for the four coordinates and experimental structure factors of the full-length rA3G have been deposited in the Protein Data Bank (PDB) with the accession numbers 6P40, 6P3Z, 6P3X, and 6P3Y. The source data underlying Figs. 2 (inset c, d), 3, 4, and Supplementary Figs. 3, 6, 7, 8, 10, and Table 3 are provided in a combined Source Data file. The datasets generated during and/or analysed during the current study are available from the corresponding author on reasonable request.

## Source Data

Source Data
