# Supplementary material for: Understanding the structural basis of HIV-1 restriction by the full length double-domain APOBEC3G
Source: Nat Commun. 2020 Jan 31;11:632. doi: 10.1038/s41467-020-14377-y (PMC6994475; doi:10.1038/s41467-020-14377-y)
Supplement: Supplementary file 1 — Supplementary information [file 41467_2020_14377_MOESM1_ESM.pdf]

Supplementary Information

Understanding the Structural Basis of HIV-1 Restriction by the Full Length Double-Domain APOBEC3G

Yang et al.

Supplementary Figures 1-10

Supplementary Tables 1-5

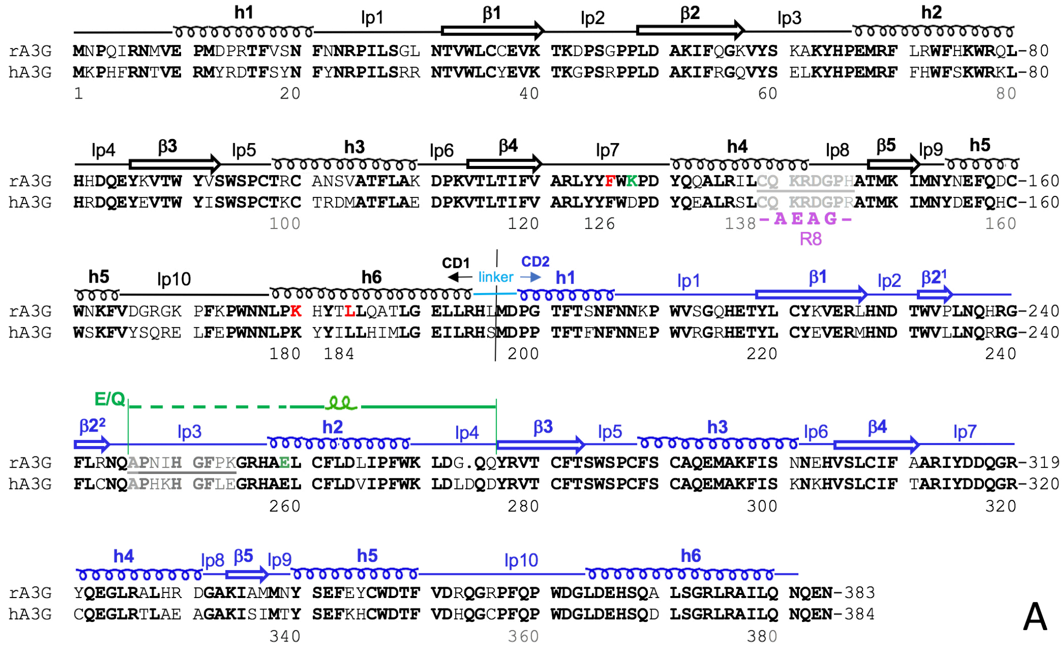

A

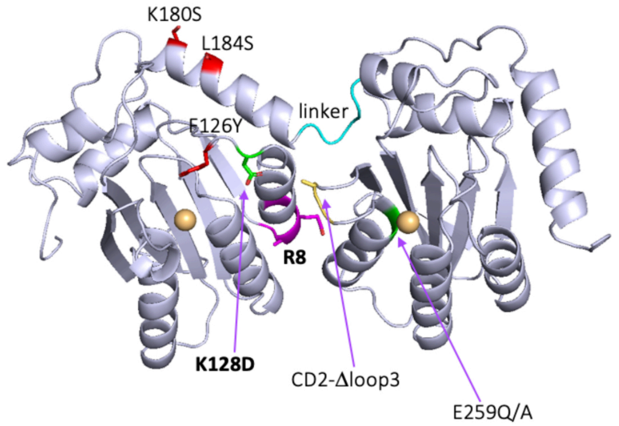

FKL structure

FKL (Crystal1)	E/Q (Crystal2)
F126Y	
K128D	K128D (to that of hA3G for Vif-degradation)
R8 (CD1 loop8 switch)	R8 (CD1 loop8 switch)
K180S/L184S	
CD2-Δloop3	
E259A	E259Q

B

Supplementary Figure 1. (A) Sequence alignment of rA3G and hA3G, with the 2nd structure assignment indicated at the top. The linker between CD1 and CD2 is indicated. The disordered loop 3 (the 9 residues in light-grey and indicated by dashed line above) and re-folded h2 (green line) of CD2 in E/Q structure are indicated. The 8 residues in CD1-loop 8 (in light-grey) are replaced by 4 residues (-AEAE-, labeled as R8 below in purple) to increase the solubility of the full-length A3G constructs for crystallization. In addition, the 2 residues in green are mutated in the E/Q structure, and the 2 residues in green plus the 3 residues in red are mutated in FKL construct (see S-Table 1). **(B)** View of the FKL structure, with the mutated residues indicated in the structure. The mutated residues in FKL and E/Q structures are listed at the bottom.

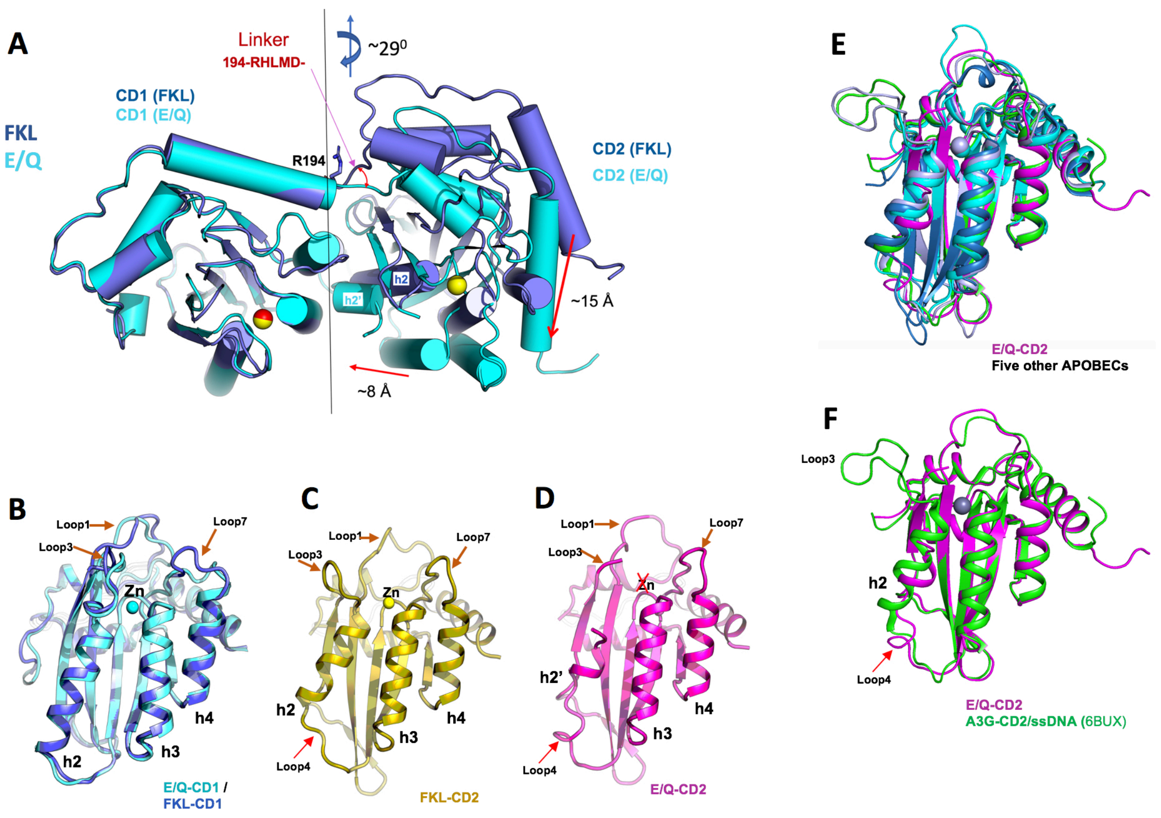

Supplementary Figure 2. Structural differences between the E/Q structure and the FKL structure of full-length rA3G. **(A)** Superimposition of the two structures of full-length rA3G. The superimposition is based on CD1 domains to reveal the rotation and angel differences between CD1 and CD2 of two structures. **(B)** The superimposition of CD1 domains of the two structures, showing close similarity. **(C)** The CD 2 domain of FKL structure. **(D)** the CD2 domain of E/Q structure. **(E)** The superimposition of E/Q CD2 (in magenta) with five other APOBEC structures, including PDBIDs 5HX4 (Zn-free A3F-CD2)[1], 5W3V (A3H)[2], 6BUX (A3G-CD2+ssDNA)[3], 3WUS (Zn-bound A3F-CD2)[4], and 3IR2 (apo-A3G structures)[5]. Except E/Q CD2, all the five other APOBEC domains compared here (including the Zn-free A3F-CD2) align with each other very well. **(F)** The superimposition of E/Q CD2 (in magenta) with the ssDNA bound A3G-CD2 (in green) (6BUX)[3]. The Zn-free E/Q CD2 does not align well in the h2-loops 3, 4 area with ssDNA bound A3G-CD2.

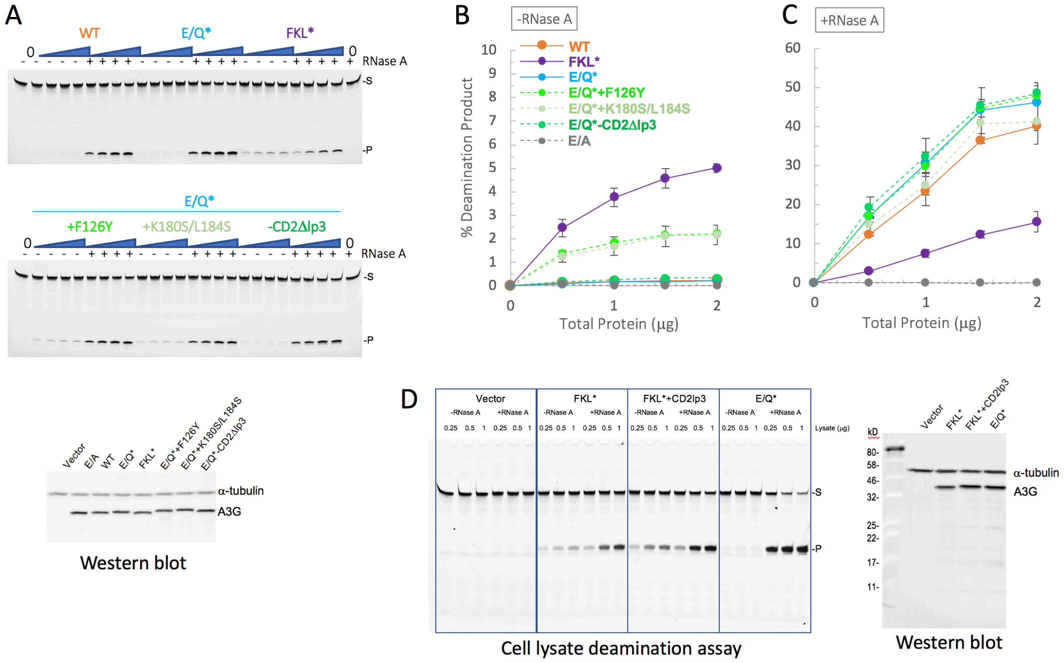

Supplementary Figure 3. Deaminase activity of the crystallized FKL and E/Q constructs with the catalytic residue E259A reverted back to E259 in FKL(designated as FKL*) and E259Q reverted back to E259 in E/Q (designated as E/Q*). **(A, B, C)** The deaminase assay results of FKL*, E/Q*, and the E/Q* construct plus additional separate mutations made in FKL. E/Q* contains WT CD2 loop3, and E/Q*-CD2Δlp3 has CD2 loop3 deleted. The assay was performed using HEK293T cell lysates expressing the proteins without (B) or with RNase A treatment (C). **(D)** The deaminase assay results of FKL*, E/Q*, and FKL*+CD2 loop3 (the original FKL and FKL* constructs contain a deleted CD2 loop3,) using cell lysates of HEK293T cells expressing the proteins. The Western blot gels in panel A and D show the protein quantity as the basis for calibrating protein concentration for the assay. Source data are provided in the Source Data file.

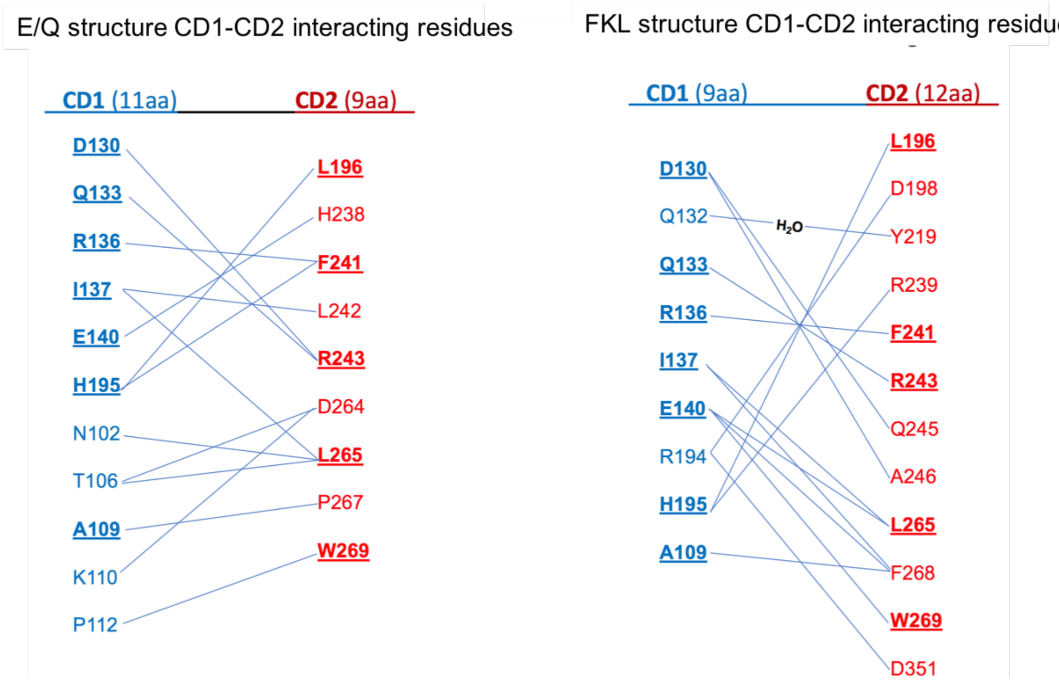

Supplementary Figure 4. The interacting residues buried within CD1-CD2 interface on CD1 or CD2 in the E/Q structure and FKL structure of full-length rA3G. The line connects the residues on the two domains indicating interaction through hydrogen bonds or H_2O mediated hydrogen bond, hydrophobic interactions, or charge-pi stacking. The residues involved in the interface interactions in both structures are underlined.

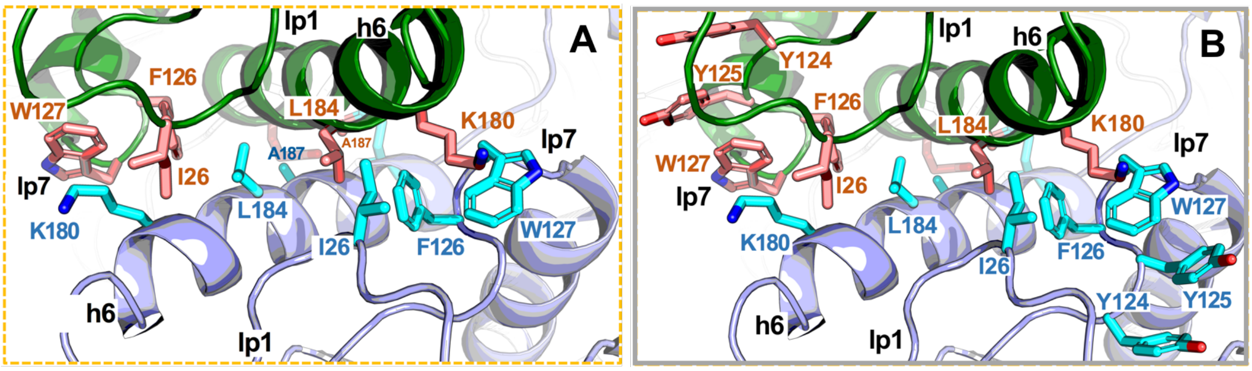

Supplementary Figure 5. The residues involved in the protein-protein dimer interface interactions. **(A)** Residues directly involved in dimer-interface hydrophobic packing, including the packing between W127 and the aliphatic side chain of K180. **(B)** The same as in panel-A, drawn with extra-residues Y125 and Y124 that packs with W127 and F126 to stabilize the conformation of W127.

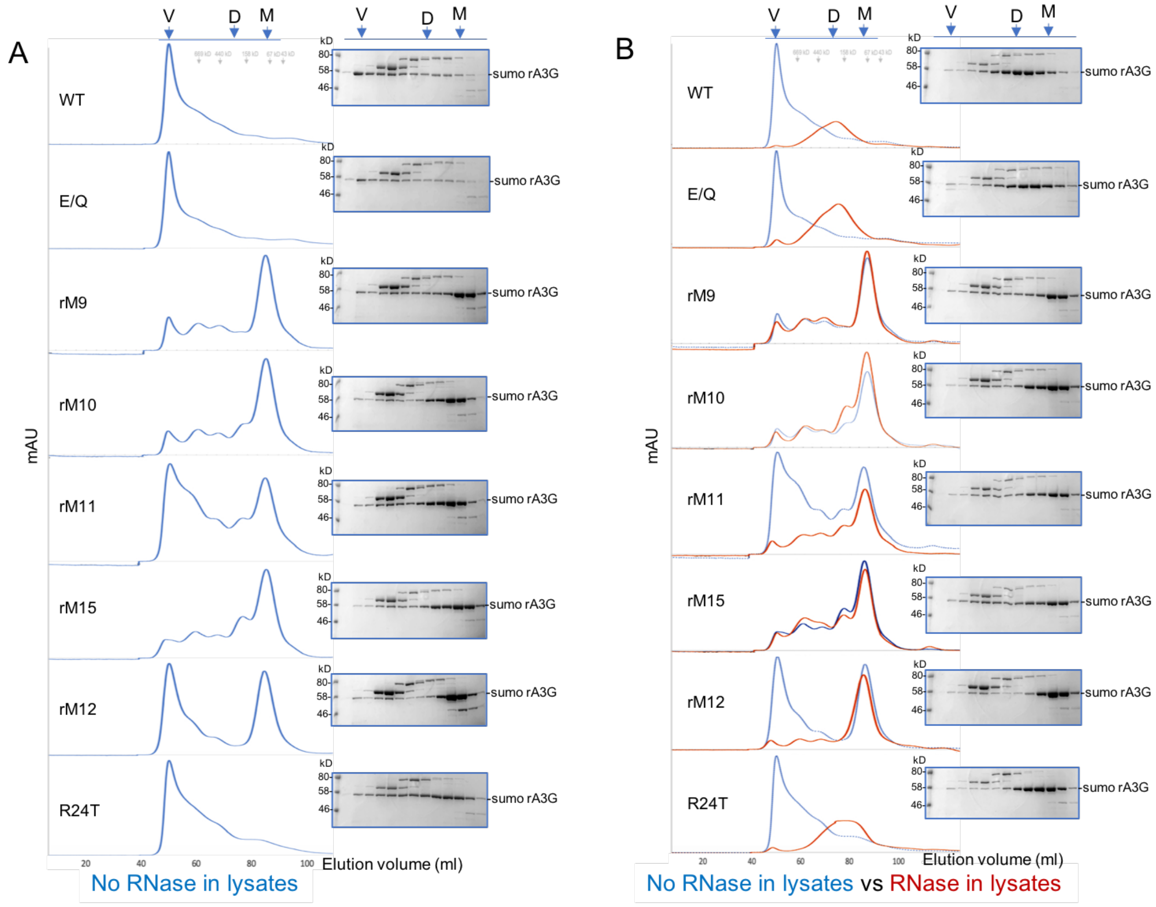

Supplementary Figure 6. (A, B) The alignment of individual SEC profiles of different mutants of full-length sumo-rA3G proteins before (A) and after (B) RNase A treatment (see Fig. 3D-E). The SDS-PAGE analysis of the protein distribution across the peak fractions is shown next to the SEC profile of each construct. Source data are provided in the Source Data file.

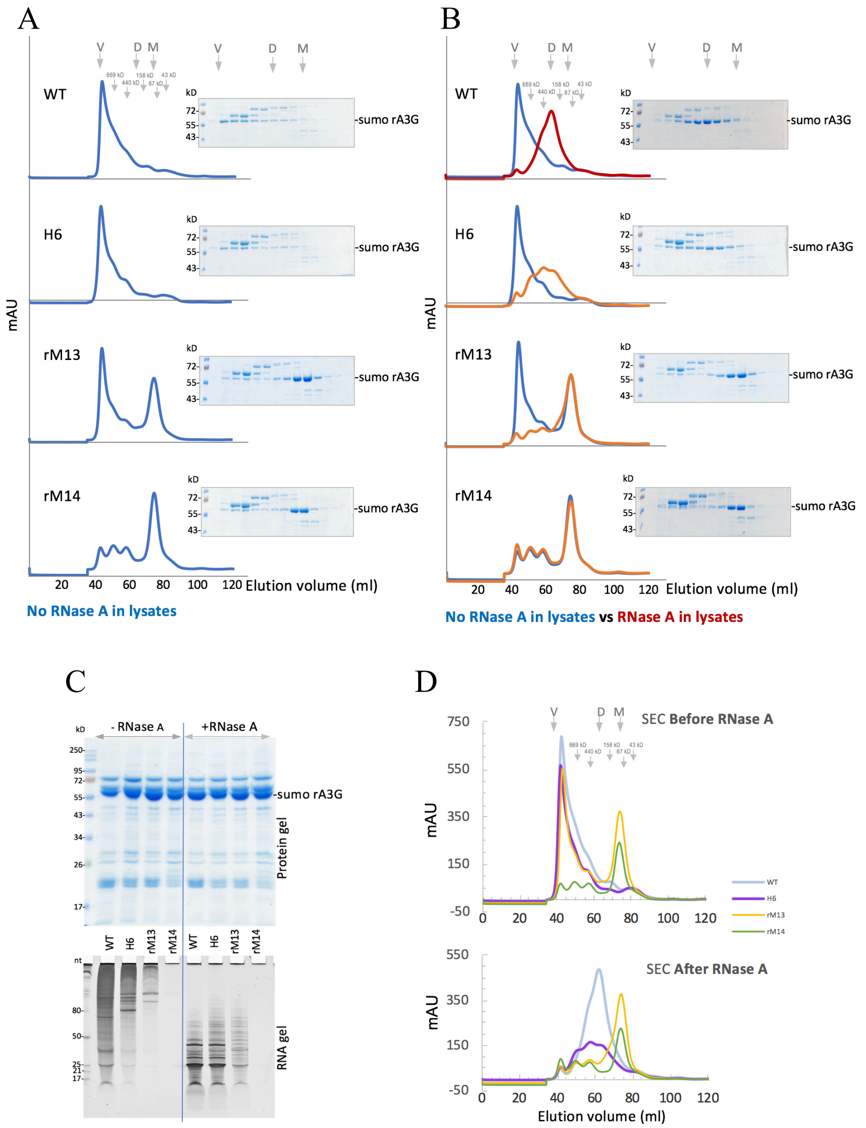

Supplementary Figure 7. Probing dimerization and RNA association of rA3G-hA3G$_{h6}$ chimera (H6) and the PEP area mutants rM13, rM14 of rA3G. The H6 chimera is based on WT rA3G but with its helix 6 replaced with the WT helix 6 sequence of hA3G so that the dimer-dimer interface residues through h6-loop1-loop7 are identical to that of hA3G. **(A, B)** The alignment of individual SEC profiles of H6 chimera and the PEP area mutants rM13, rM14 of full-length rA3G proteins before (A) and after (B) RNase A treatment. WT rA3G is used as a control. The SDS-PAGE analysis of the protein distribution across the peak fractions is shown next to the SEC profile of each construct. All constructs were assayed as His$_6$-sumo-fusions. **(C)** The SDS-PAGE protein gel analysis (top) of the His$_6$-sumo-rA3G WT and various mutants after nickel affinity column purification, and 20% denaturing urea polyacrylamide gel analysis (bottom) of RNAs associated with the proteins without/with RNase A treatment during purification. **(D)** Superdex-200 size exclusion chromatography (SEC) analysis of the proteins before (top) and after (bottom) RNase A treatment. The positions corresponding to void volume, dimer, and monomer are indicated with arrows. Source data are provided in the Source Data file.

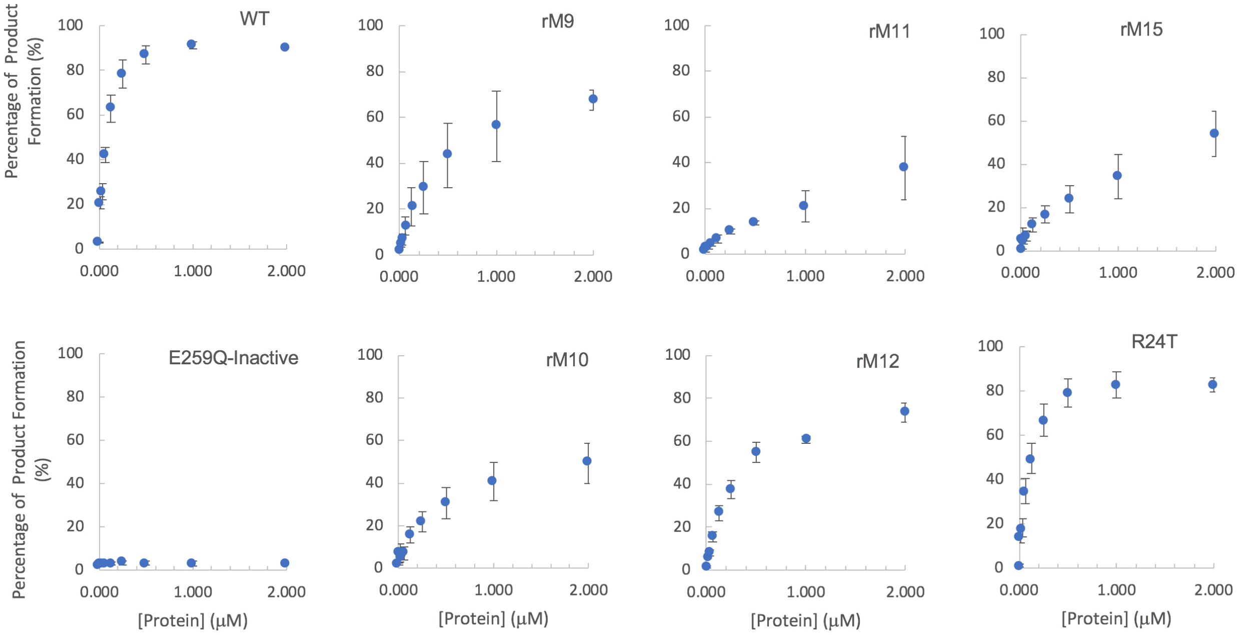

Supplementary Figure 8. Deaminase activity of rA3G mutant proteins (see S-Table 3) purified from E. coil expression. WT and E/Q (E259Q) were used as positive and negative controls. Source data are provided in the Source Data file.

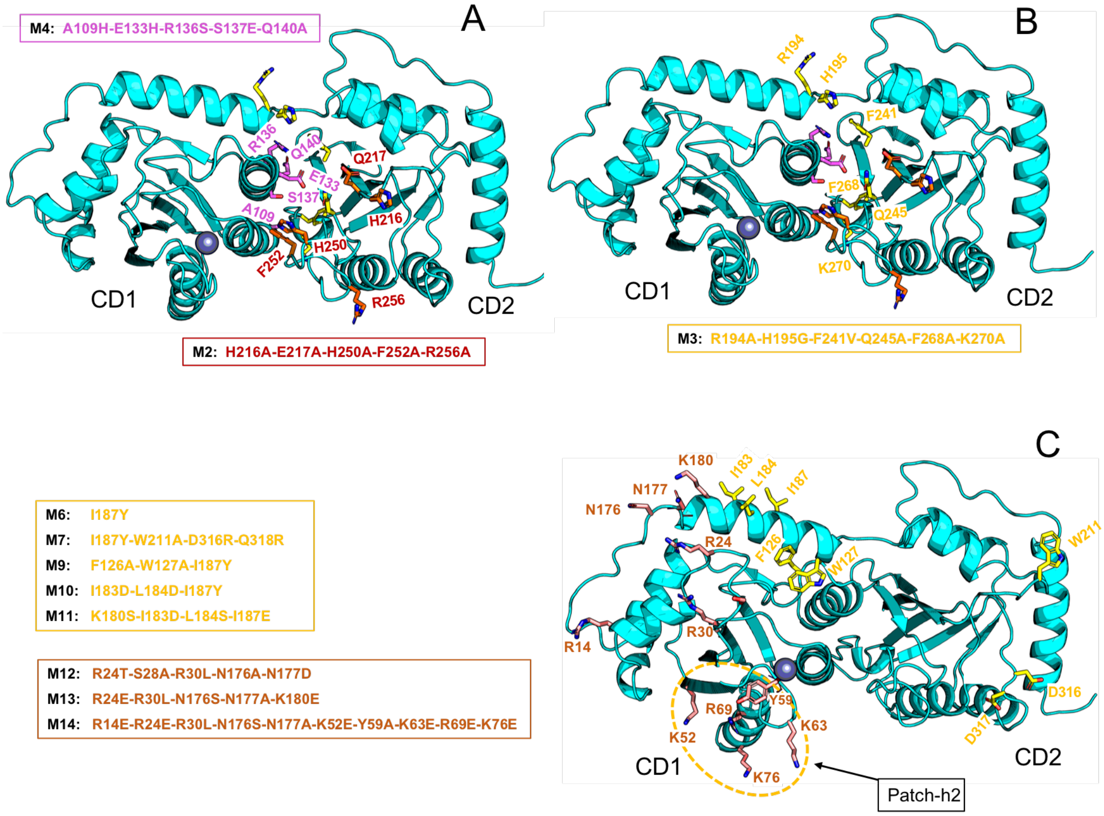

Supplementary Figure 9. The ribbon representation of hA3G model built based on rA3G E/Q structure, with all mutated residues shown in stick model and labeled. **(A, B).** Locations of all the mutated residues in the hA3G mutants M2, M3, and M4 in both panels-A and B, but the M2 residues (in red) and M4 (in pink) are labeled in panel-A, and M3 (in yellow) labeled in panel-B. **(A)** The mutated residues of M2 are on CD2-loop 1 and 3 that are near CD1 but surface exposed. The mutated residues of M4 are on the CD1 side within the interface area with CD2. **(B)** The mutated residues of M3 are on the CD2 side on the interface area with CD1. **(C).** Locations of all the mutated residues in hA3G mutants M6, M7, M9, M10, and M11 (side chains colored in yellow), and the mutants M12, M13, and M14 (colored in salmon). The four K/R residues on the patch centered round CD1 h2 (Patch-h2) included only in M14 is indicated by a yellow circle. It is worth noting that despite the conformational difference between the E/Q and the FKL structures mostly at the CD2 Zn-center and at the interface between CD1-CD2, the location of these surface residues for mutation are generally conserved on both structures. Therefore we only use the E/Q for hA3G model building.

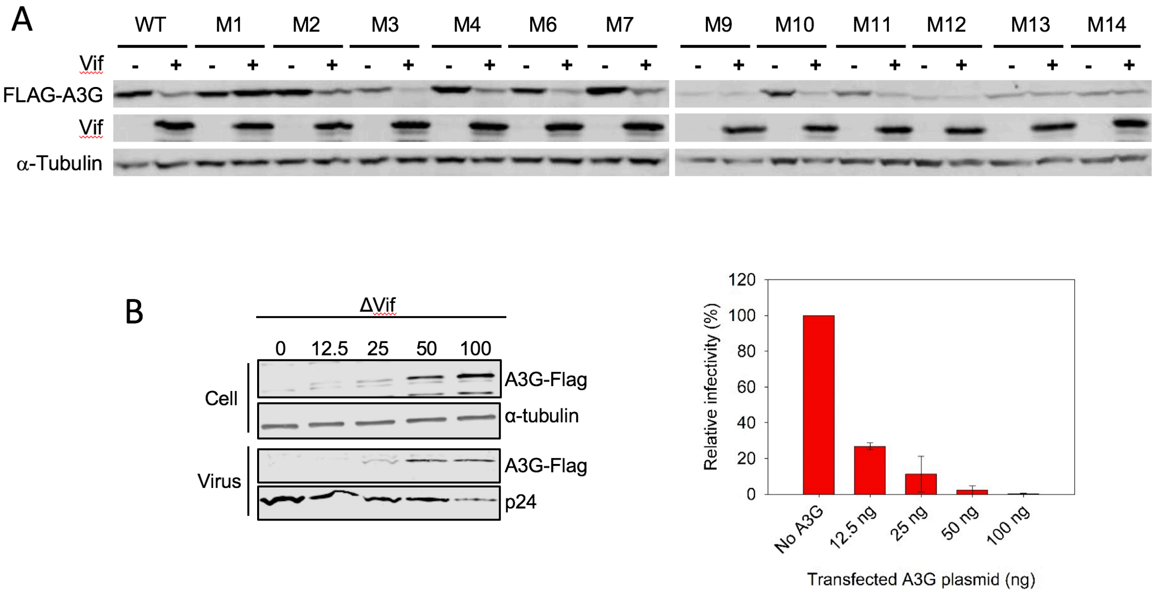

Supplementary Figure 10. HIV-1 Vif sensitivity of hA3G mutants and Titration of WT hA3G expression level and HIV-restriction activity. **(A).** Western blot showing HIV-1 Vif sensitivity assay of the hA3G mutants (see S-table 4). The presence and absence of Vif is indicated. The detectible protein of M9 and M12-M14 constructs appeared to be at low steady state levels even if with increasing amount of transfected DNA after multiple tests with side-side comparison with WT. **(B).** Left-panel, reducing the transfected WT A3G plasmid DNA from 100 ng to 12.5 ng to lower the expression level in order to achieve comparable protein levels as observed for M12-M14. Right-panel, the relative HIV infectivity at different WT A3G levels, showing that at 25-50 ng plasmid DNA that show comparable protein expression to M12-M14, WT A3G showed approximately 4-fold higher restriction activity than M12-M14 (see Fig. 4A-C). Source data are provided in the Source Data file.

Supplementary Table 1. Mutations on E/Q and FKL constructs for FL rA3G structure. Besides K128D and the catalytic E259Q mutation, E/Q construct only had a 4-residue (-AEAG-) replacement for the 8-residue (139-CQKRDGPH-146) within CD1 loop 8. FKL construct has additional F126/Y/K180S/L184S and an 8-residue deletion within CD2-loop 3.

rA3G	CD1				CD2		Crystals resolution (Å)
**WT**	**F126**	**K128**	(CD1-loop 8) **139-CQKRDGPH-146**	**K180-L184**	(CD2-loop 3) **247-PNIHGFPK-254**	**Catalytic E259**	
**E/Q**	–	K128D	-AEAG-	–	–	E259Q	[a]2.40; 2.58; 2.85
**FKL**	F126Y	K128D	-AEAG-	K180S/L184S	Deleted (Δloop3)	E259A	[b]2.47

Note:

[a] Crystals obtained from three conditions having the same dimeric structure, but different resolution.
2.40 Å data from 50 mM HEPES pH 7.0, 10 mM $MgCl_2$, 1.5 M $(NH4)_2SO_4$, protein buffer in HEPES, pH 7.5.
2.58 Å data from 50 mM Tris-Cl pH 7.4, 25 mM $MgCl_2$, 2.0 M $(NH4)_2SO_4$, protein buffer in Tris-Cl pH 8.0;
2.85 Å data from 50 mM MES, pH 5.2, 10 mM $MgCl_2$, 1.8 M $LiSO_4$, and protein buffer in HEPES at pH 7.5.

[b] 2.47 Å data from 0.1 M MES, pH 6.9, 8% PEG 20K, protein buffer in HEPES at pH 7.5.

Supplementary Table 2. Data collection and refinement statistics of full length rA3G structures

	**FKL (6P40)**	**E/Q (pH 5.2) (6P3Z)**	**E/Q (pH 7.0) (6P3X)**	**E/Q (pH 7.4) (6P3Y)**
		**Data collection**		
Space group	P 1 21 1	P 1 21 1	P 1 21 1	P 1 21 1
Cell dimensions				
a, b, c (Å)	62.1, 86.4, 90.2	67.8, 153.5, 67.8	67.5, 153.2, 67.5	67.7, 153.9, 67.7
α, β, γ (°)	90.0, 101.4, 90.0	90.0, 114.1, 90.0	90.0, 114.9, 90.0	90.0, 116.4, 90.0
Resolution (Å)	50-2.47 (2.51-2.47)*	50-2.85 (2.95-2.85)	50-2.40 (2.49-2.40)	50-2.58 (2.67-2.58)
Rsym or Rmerge	0.10 (0.64)	0.21 (0.67)	0.10 (0.43)	0.13 (0.51)
I/σI	15.3 (3.2)	8.7 (2.1)	11.9 (3.4)	10.9 (3.7)
Completeness (%)	99.5 (98.6)	94.3 (75.7)	97.8 (84.8)	97.6 (82.4)
Redundancy	3.9 (3.6)	6.7 (4.7)	6.7 (5.8)	6.6 (5.7)
No. reflections	65342	27963	47319	38528
		**Refinement**		
Rwork/Rfree	17.29/22.84	20.41/24.59	18.86/23.70	19.56/23.75
No. atoms	6155	6044	6240	6157
Protein	6040	6042	6042	6042
Ligand/ion	4	2	2	2
Water	111	--	196	113
B-factors	50.02	44.30	53.12	55.51
Protein	50.08	44.31	53.29	55.69
Ligand/ion	46.97	35.64	40.41	42.83
Water	47.02	--	47.99	45.95
r.m.s. deviations				
Bond lengths (Å)	0.009	0.004	0.004	0.005
Bond angles (°)	1.059	0.811	0.739	0.935

Highest-resolution shell is shown in parentheses.

Supplementary Table 3. The *in vitro* studies of mutants of FL rA3G designed either to disrupt dimer-interface interactions or to disrupt the enhanced positive electrostatic potential surface to disrupt RNA-binding at the dimer junction area.

rA3G	CD1			CD2	50nt RNA binding (Kd, nM)	50nt DNA binding (Kd, nM)
**WT** [a]	R8[b]	K128D[c]		E259[d]	22 ± 7	24 ± 4
**E/Q**	R8	K128D		E259Q	19 ± 5	10 ± 2
**rM9**	R8	K128D	F126A-W127A-A187Y [Within dimer-interface]	E259	20 ± 4	74 ± 14
**rM10**	R8	K128D	T183D-L184D-A187Y [Within dimer-interface]	E259	24 ± 6	53 ± 8
**rM11**	R8	K128D	K180S-T183D-L184S-A187E [Within dimer-interface]	E259	21 ± 5	89 ± 23
**rM15**	R8	K128D	I26A-K180S-L184S-A187E [Within dimer-interface]	E259	21 ± 3	71 ± 13
**rM12**	R8	K128D	R24T-S28A-N176A-N177D [PEP surface at dimer junction]	E259	52 ± 11	188 ± 27
**rM13**	R8	K128D	R24E-N176A-N177A-K180E [PEP surface at dimer junction]	E259	43 ± 9	155 ± 36
**rM14**	R8	K128D	R15E-R24E-N176S-N177A-K52E-Y59A-K63E-R69E-K76E [PEP surface plus positive patch]	E259	15 ± 4	102 ± 18
**R24T**	R8	K128D	R24T	E259	21 ± 6	51 ± 8
**H6 chimera** [c]	R8	K128D	rA3G 181-HYTLLQAT replaced by hA3G 181-YYILLHIM	E259	ND	ND

Note: Mutants at the buried dimer-interface mutants: rM10, rM11, rM15. Mutants on the enhanced positive electrostatic potential: rM12. [a] WT: this designated WT, a parental construct for other full-length rA3G mutants, contains a K128D that enables rA3G to be sensitive to HIV-1 Vif mediated degradation and a replaced CD1-loop 8 (R8, see note [b] next). [b] R8 (replaced loop 8): A stretch of 8 residues 139-CQKRDGPH-146 within CD1-loop 8 is replaced by 4 residues -AEAG- from the CD2-loop 8 of hA3G to increase solubility of the full-length construct. [c] E259 is essential for deaminase activity and E259Q is catalytically inactive. [c] H6 chimera: rA3G containing the helix 6 sequence of hA3G CD1, this H6 construct has the exact hA3G residues at the protein-protein dimer interface if hA3G dimerizes in the same way as rA3G.

All mutated residues except those in red are identical in rA3G and hA3G. The two non-identical residues have matching residues in hA3G as follows (rA3G/hA3G): A187/I187, T183/I183 (see S-Fig. 1). The Kd values for RNA and ssDNA binding were estimated based on native gel shift assay (see S-Fig. 7).

The Kd for RNA or ssDNA binding of the purified proteins of different mutants were estimated using native gel shift assay (see Methods). Source data are provided in the Source Data file.

Supplementary Table 4. The in-cell studies of full-length hA3G mutants designed either to disrupt dimer-interface (DI) interactions or to disrupt the enhanced positive electrostatic potential (PEP) surface to disrupt RNA-binding at the dimer junction area.

hA3G	Mutations of hA3G	Notes
**WT**		
**M1**	D128K	
**M2**	H216A-E217A-H250A-F252A-R256A	Mutations on CD2 loop-1, 3 adjacent to CD1
**M3**	R194A-H195G-F241V-Q245A-F268A-K270A	Mutations on CD2 interface with CD1
**M4**	A109H-E133H-R136S-S137E-Q140A	Mutations on CD1 interface with CD2
**M6**	I187Y	Dimer interface mutation on CD1 h6
**M7**	I187Y-W211A-D316R-Q318R	Dimer interface mutation on CD1 h6 plus mutations on CD2 loop 1, 7
**M9**	F126A-W127A-I187Y	Mutation on CD1 loop 7 (W127) plus CD1 h6
**M10**	I183D-L184D-I187Y	Dimer interface mutation on CD1 h6
**M11**	K180S-I183D-L184S-I187E	Dimer interface mutation on CD1 h6
**M12**	R24T-S28A-R30L-N176A-N177D	PEP surface mutations
**M13**	R24E-R30L-N176S-N177A-K180E	PEP surface mutations
**M14**	R14E-R24E-R30L-N176S-N177A -K52E-Y59A-K63E-R69E-K76E	PEP surface mutations plus positive patch

Note: All mutated residues except those in red are identical in rA3G and hA3G. The non-identical residues in red shown for hA3G here have matching residues in rA3G as follows (hA3G/rA3G): R30/L30, E133/Q133, S137/I137, I187/A187, I183/T183 (see S-Fig. 1). Positive EP: Positive electrostatic potentials that are enhanced at the dimer junction.

Supplementary Table 5. Analysis of mutagenesis in HIV-1 proviral DNA induced by hA3G mutants in the absence of Vif.

A3 enzyme	Base pairs sequenced	G→A mutations (total)	GG→AG mutations (total)	G→A mutations (per kb)	GG→AG mutations (per kb)
No A3G	2808	19	14	6.8	5.0
A3G-WT	2106	41	33	19	16
M1 (D128K)	3861	57	44	15	11
M9	3159	32	25	10	7.9
M10	2457	21	16	8.5	6.5
M11	3159	35	28	11	8.9
M12	2106	21	19	10	9.0
M13	4212	55	44	13	10
M14	3510	20	15	5.7	4.3

A 351 bp region of HIV-1 *protease* was PCR amplified from a single-cycle replication assays. Clones were sequenced, aligned with Clustal Omega (Sievers et al., 2011), and analyzed using Hypermut (Rose and Korber, 2000). Mutations not in the GG→AG context were GA→AA or GC→AC.

Supplementary references:

1 Shaban, N. M., Shi, K., Li, M., Aihara, H. & Harris, R. S. 1.92 Angstrom Zinc-Free APOBEC3F Catalytic Domain Crystal Structure. *Journal of molecular biology* **428**, 2307-2316, doi:10.1016/j.jmb.2016.04.026 (2016).

2 Bohn, J. A. *et al.* APOBEC3H structure reveals an unusual mechanism of interaction with duplex RNA. *Nature communications* **8**, 1021, doi:10.1038/s41467-017-01309-6 (2017).

3 Maiti, A. *et al.* Crystal structure of the catalytic domain of HIV-1 restriction factor APOBEC3G in complex with ssDNA. *Nature communications* **9**, 2460, doi:10.1038/s41467-018-04872-8 (2018).

4 Nakashima, M. *et al.* Structural Insights into HIV-1 Vif-APOBEC3F Interaction. *Journal of virology* **90**, 1034-1047, doi:10.1128/JVI.02369-15 (2016).

5 Shandilya, S. M. *et al.* Crystal structure of the APOBEC3G catalytic domain reveals potential oligomerization interfaces. *Structure* **18**, 28-38, doi:10.1016/j.str.2009.10.016 (2010).
