# Supplementary material for: Understanding the structural basis of HIV-1 restriction by the full length double-domain APOBEC3G
Source: Nat Commun. 2020 Jan 31;11:632. doi: 10.1038/s41467-020-14377-y (PMC6994475; doi:10.1038/s41467-020-14377-y)
Supplement: Supplementary file 3 — Reporting Summary [file 41467_2020_14377_MOESM3_ESM.pdf]

nature research

Corresponding author(s): Xiaojiang Chen

Last updated by author(s): 11/28/2019

Reporting Summary

Nature Research wishes to improve the reproducibility of the work that we publish. This form provides structure for consistency and transparency in reporting. For further information on Nature Research policies, see Authors & Referees and the Editorial Policy Checklist.

Statistics

For all statistical analyses, confirm that the following items are present in the figure legend, table legend, main text, or Methods section.

n/a	Confirmed	
☐	☒	The exact sample size (n) for each experimental group/condition, given as a discrete number and unit of measurement
☐	☒	A statement on whether measurements were taken from distinct samples or whether the same sample was measured repeatedly
☒	☐	The statistical test(s) used AND whether they are one- or two-sided<br>*Only common tests should be described solely by name; describe more complex techniques in the Methods section.*
☒	☐	A description of all covariates tested
☒	☐	A description of any assumptions or corrections, such as tests of normality and adjustment for multiple comparisons
☒	☐	A full description of the statistical parameters including central tendency (e.g. means) or other basic estimates (e.g. regression coefficient) AND variation (e.g. standard deviation) or associated estimates of uncertainty (e.g. confidence intervals)
☒	☐	For null hypothesis testing, the test statistic (e.g. F, t, r) with confidence intervals, effect sizes, degrees of freedom and P value noted<br>*Give P values as exact values whenever suitable.*
☒	☐	For Bayesian analysis, information on the choice of priors and Markov chain Monte Carlo settings
☒	☐	For hierarchical and complex designs, identification of the appropriate level for tests and full reporting of outcomes
☒	☐	Estimates of effect sizes (e.g. Cohen's d, Pearson's r), indicating how they were calculated

Our web collection on statistics for biologists contains articles on many of the points above.

Software and code

Policy information about availability of computer code

Data collection	Crystallography data were collected and processed using synchrotron X-ray beams and standard softwares. No software was used for the biochemical and functional data collection of this study.
Data analysis	N/A

For manuscripts utilizing custom algorithms or software that are central to the research but not yet described in published literature, software must be made available to editors/reviewers. We strongly encourage code deposition in a community repository (e.g. GitHub). See the Nature Research guidelines for submitting code & software for further information.

Data

Policy information about availability of data

All manuscripts must include a data availability statement. This statement should provide the following information, where applicable:

- Accession codes, unique identifiers, or web links for publicly available datasets
- A list of figures that have associated raw data
- A description of any restrictions on data availability

The authors declare that the data supporting the findings of this study are available within the paper and its Supplementary Information files and Data source files, also available from the corresponding author upon reasonable request. The PDB ID numbers have been provided in the paper, and accessible through Protein Data Bank (PDB) upon publication.

Field-specific reporting

Please select the one below that is the best fit for your research. If you are not sure, read the appropriate sections before making your selection.

☒ Life sciences ☐ Behavioural & social sciences ☐ Ecological, evolutionary & environmental sciences

For a reference copy of the document with all sections, see nature.com/documents/nr-reporting-summary-flat.pdf

Life sciences study design

All studies must disclose on these points even when the disclosure is negative.

Sample size	No statistical analysis was carried out.
Data exclusions	No data is excluded.
Replication	All experiments were successfully replicated at least three times.
Randomization	Not applicable.
Blinding	Not applicable.

Reporting for specific materials, systems and methods

We require information from authors about some types of materials, experimental systems and methods used in many studies. Here, indicate whether each material, system or method listed is relevant to your study. If you are not sure if a list item applies to your research, read the appropriate section before selecting a response.

Materials & experimental systems

n/a	Involved in the study	
☐	☒	Antibodies
☐	☒	Eukaryotic cell lines
☒	☐	Palaeontology
☒	☐	Animals and other organisms
☒	☐	Human research participants
☒	☐	Clinical data

Methods

n/a	Involved in the study	
☒	☐	ChIP-seq
☒	☐	Flow cytometry
☒	☐	MRI-based neuroimaging

Antibodies

Antibodies used	anti-HIV-1 Vif monoclonal antibody, NIH AIDS Reagent Program (lot #319); anti-FLAG M2 mAb from mouse (F3165, Sigma); anti-a-tubulin mAb from mouse (GT114, GeneTex); Cy3-labelled goat-anti-mouse mAb (PA43009, GE Healthcare); Licor IRDye antibodies produced in goat (IRDye 680-labeled anti-rabbit, 926-68071) and (IRDye 800-labeled anti mouse, 926-32210).
Validation	anti-HIV-1 Vif monoclonal antibody was provided from the NIH AIDS Reagent Program (lot #319); Mouse anti-FLAG monoclonal antibody is the commonly used one that was purchased from Sigma. All antibodies where shown to be specific against the advertised targets.

Eukaryotic cell lines

Policy information about cell lines

Cell line source(s)	293T (or HEK293T) Cells from ATCC
Authentication	N/A
Mycoplasma contamination	No contamination
Commonly misidentified lines (See ICLAC register)	N/A
