# Supplementary material for: Understanding the structural basis of HIV-1 restriction by the full length double-domain APOBEC3G
Source: Nat Commun. 2020 Jan 31;11:632. doi: 10.1038/s41467-020-14377-y (PMC6994475; doi:10.1038/s41467-020-14377-y)
Supplement: Supplementary file 2 — Peer Review [file 41467_2020_14377_MOESM2_ESM.pdf]

Reviewers' comments:

Reviewer #1 (Remarks to the Author):

Yang and co-workers present crystal structures of rhesus macaque A3G (rA3G) variants. Major findings are inter-domain orientation of the N-terminal and C-terminal domain, and positively charged surface appeared at the inter-molecular interface formed by two N-terminal domains. This positively charged surface binds RNA, which stabilizes the rA3G dimer formation. Yet, neither dimerization nor the RNA-binding surface is essential for viral incorporation and HIV-1 restriction.

This manuscript provides the first experimentally determined structure of double domain APOBEC3 proteins. It is highly significant achievement because double domain APOBEC3 proteins are insoluble which has hampered structural study for more than 10 years. That being said, the manuscript needs to be improved by including experimental data and/or discussion regarding ssDNA substrate binding and Vif binding because the biological significance of the rA3G dimer structure is somewhat diminished since dimerization and the RNA binding region found at the dimerization interface were not critical for virion encapcidation nor HIV-1 restriction by human A3G (hA3G).

Major concerns:
1. Huthoff et al. (PLoS Pathog. 2009) have previously proposed a model of human A3G dimer formed by interaction of two N-terminal domains. Although Huthoff's structural model of hA3G dimer was significantly different from the structure of rA3G dimer, the hA3G dimer model showed some similarities as Y124 and W127 formed an inter-molecular hydrophobic core, and R24 was a part of positively charged inter-molecular interface. Huthoff et al. mutated R24 of hA3G, and observed substantial reduction of both virion encapsidation and HIV-1 restriction (Fig. 6 of Huthoff et al.). These results are substantially different from the results shown in Fig. 4 of this manuscript since disruption of the RNA-binding region containing R24 of hA3G was not critical for virion encapsidation nor HIV-1 restriction. It is appropriate to refer the paper by Huthoff et al , and address those controversial results.

2. Structure of the rA3G E/Q variant may not be biologically relevant for hA3G because Zn is essential for the catalytic reaction, and HIV-1 restriction by hA3G is predominantly achieved through deamination of cytidines in viral DNA.

3. In the rA3G FKL structure, the N-terminal and C-terminal domains interact through h3, h4 and loop7 of the N-terminal domain and h1, h2 and β2 and loop3 of the C-terminal/catalytic domain. Since h1, h2 and β2 and loop3 are involved to form the catalytic pocket, the FKL rA3G in the provided structure is likely to have reduced catalytically activity. Does the rA3G FKL structure represent a catalytically active intra-molecular domain orientation?

4. The dimer interface found in the full-length rA3G has been already reported by the same laboratory as they have published crystal structure of the N-terminal domain of rA3G (Xiao et al., Nat Commun. 2016). The Xiao et al. paper also discussed the nucleic acid binding at the dimer interface. Authors should emphasize what is new in the full-length rA3G structure.

5. rA3G dimer is more stable in solution (Xiao et al., Nat Commun. 2016 and this manuscript) than hA3G dimer since hA3G is most likely under equilibrium of various multimers even after RNAse treatment and size-exclusion column separation (e.g. Iwatani et al., J Virol, 2006; Chelico et a., Nat Stuc. Mol. Biol, 2006; Morse et al., Nat. Commun. 2017). Therefore, oligomeric states of rA3G and hA3G may be different in solution. Extrapolation of dimeric structure of rA3G to hA3G should be considered more carefully since it may or may not be true in hA3G.

Reviewer #2 (Remarks to the Author):

This manuscript reported a crystal structure of full-length rhesus macaque A3G. The structure revealed a detailed arrangement of CD1 and CD2, the structural basis for A3G RNA binding and dimer formation. Based on mutagenesis studies, the authors argued that the disruption of the PEP surface in the dimeric interface disrupted RNA binding and A3G dimerization, but had no interference on A3G encapsidation and HIV restriction. Solving the full-length A3G structure is a breakthrough in the A3G research field and will have many important impacts in the area of A3G biology research. While this manuscript is leading in solving the full-length structure, the reviewer has following questions/concerns that should draw the authors' attention.

1. In order to solubilize and crystallize rA3G, several mutations were introduced into the rA3G protein. The eight amino acids, CQKRDGPH, were replaced by four amino acids, AEAE, in the h4 and lp8 region of CD1 (S-Fig. 1). The crystal structures showed that this region was directly involved in the interaction between CD1 and CD2. The E140 interacted with amino acid(s) in CD2 (S-Fig. 3). It raises the reviewer's concern that the mutation might alter the interaction between CD1 and CD2. The authors should address the concern in the discussion.

2. The mutations introduced in hA3G was based on the assumption that rA3G and hA3G have a similar crystal structure. It is plausible to accept this assumption given the high homologous amino acid sequences between them. However, there are some different amino acid residues involved in the interface between rA3G and hA3G as highlighted in red(S-Table 3 and S-Table 4). Therefore, the reviewer thinks it is essential for the authors to provide experimental evidence to confirm the mutants' dimer formation or the RNA bindings as they claimed in the manuscript.

3. On page 10, the authors claimed that "….. the residues mutated in M12-14 to disrupt RNA binding in the PEP and nearby areas on CD1 are not critical for HIV-1 restriction and virion encapsidation,……". What the reviewer saw was that the M12-M14 all had dramatic packaging defect comparing to WT and M1 in Fig 4A. The authors should repeat this experiment with a similar expression level of all A3G proteins. If it is not possible to enhance M12-14 expression, what about reducing other protein expression? Again, the binding of M12-M14 with RNA needs to be confirmed by experiment.

4. minor issues:

Western blot of cell lysates and virion should be labeled in Fig 4 A and B

On page 9, 3rd line in the last paragraph, "M2 and M4 both had significantly lower HIV-1…." should be "M2 and M3 both….."?

Reference 44 has been retracted.

Reviewer #3 (Remarks to the Author):

In the manuscript, Yang et al. present their work on determining the crystal structure of a full-length two-domain A3 protein, Macaca mulatta A3G. They find that the two domains are flexible around a five amino acid linker, with one conformation contorts the fold of the catalytically active domain. They show that a positive electrostatic patch is formed at the dimer interface and is involved in RNA binding, and disrupting RNA binding and oligomerization can be separated from packaging into HIV virions and restriction of HIV. This work is an important advancement for the field of A3 structural studies, as well as HIV research. However, the manuscript would be strengthened if the authors addressed the following concerns:

- Substantial impact in presentation is lost due to the design of the figures. The authors mentioned that they had screened A3G homologs from different primate species and found two engineered rA3G FL constructs with good behavior for structural studies. These mutations should be clearly marked in the structural figures. It would also be very helpful to include a description of the rationales for the design of those mutants, especially the FKL and E/Q mutants which gave the crystal structures, so readers can understand how these crystal structures may vary from the WT rA3G protein.
- In Figure 1A & B, it is important to highlight the short liner region connecting CD1 and CD2. In Figure 1D, label h1, β2, loop 3 and 7 which are involved in the CD1-CD2 interaction; Similarly, in Figure 1E, label h6, β2, loop4 and 10.
- Are the differential packing orientations between E/Q and FKL mutants caused by the intrinsic plasticity of the FL rA3G or affected by the mutations introduced (which may lead to different crystal packing interactions)?
- At the end of the first results section, the authors mentioned that the E/Q CD2 has a much closer packing interaction with CD1, but the buried surface areas are quite similar (623 vs. 700). It would be helpful to elaborate this more.
- The E/Q structure seems less than likely to represent the deamination competent state since it has kicked out the zinc in its catalytic pocket, does this mean that the conformation of the FKL structure is more biologically relevant? Some discussions would benefit the readers.
- It would be interesting to expand more on Figure 3B, rM11? It seems surprising that it still bound RNA when the other interface mutants that only had one different mutation did not bind any RNA at all.
- In figure 4, was the low expression of M11-M14 taken into account in the infectivity assays? Would those mutants restricted HIV infection to the same level as WT if they had been expressed or packaged at the same amount?
- Figure 4D was first mentioned as a deamination activity result, it does not seem to be correct. Should be Fig 4E?
- In figure 4E, the deaminase activity of M1 seems to be omitted. Also, were these deamination levels normalized to the expression level of the protein?
- There is only one residue in green in S-Fig.1, but the caption says "2 residues in green color are mutated".
- For S-Figure 6 and S-Table 3 – the values generated from the EMSA experiments are overly precise with many digits of significant figures and have no errors associated with them. Were these experiments performed in triplicate? More statistical rigor would be needed.
- For S-Table 3, some numbers are with too many digits of significant figures, such as cell parameters, I/SigI, and B-factors. The resolution cutoffs are too conservative: I/SigI from 2.1 to 3.7. Higher resolution data can be included to improve the precision of the structure. The use of CC1/2 values has justified the inclusion of more high-resolution, weak data (sometimes with I/SigI <1) in refinement (see Karplus et al. Science 2012, doi: 10.1126/science.1218231). Although the quality of the reported structure is acceptable and the potential additional improvement is not expected to change any major conclusions in the manuscript, a more precise model can serve better for those in the community who may use the structural model to facilitate their research.
- It would be interesting to see an overlay between the E/Q CD2 domain and other A3 structures – such as PDBIDs 5HX4, 5W3V, 6BUX, and any of the A3G CD2 structures.

Point-by-point response:

We thank the reviewers and editor for the valuable time/efforts and very insightful and constructive comments about our manuscript. We have added critical new data, and have revised the manuscript based on the suggestion of the review comments. A detailed point-by-point response is described below.

Reviewer #1 (Remarks to the Author):

Yang and co-workers present crystal structures of rhesus macaque A3G (rA3G) variants. Major findings are inter-domain orientation of the N-terminal and C-terminal domain, and positively charged surface appeared at the inter-molecular interface formed by two N-terminal domains. This positively charged surface binds RNA, which stabilizes the rA3G dimer formation. Yet, neither dimerization nor the RNA-binding surface is essential for viral incorporation and HIV-1 restriction.

This manuscript provides the first experimentally determined structure of double domain APOBEC3 proteins. It is highly significant achievement because double domain APOBEC3 proteins are insoluble which has hampered structural study for more than 10 years. That being said, the manuscript needs to be improved by including experimental data and/or discussion regarding ssDNA substrate binding and Vif binding because the biological significance of the rA3G dimer structure is somewhat diminished since dimerization and the RNA binding region found at the dimerization interface were not critical for virion encapcidation nor HIV-1 restriction by human A3G (hA3G).

RESPONSE: We greatly appreciate the overall positive and fair reviews and constructive comments from this reviewer. Regarding the issue on ssDNA binding and Vif binding, we revised the manuscript hoping to have a better presentation and discussion on these aspects in this revision.

1) We added new data in S-Fig. 8 about in vitro deaminase activity of purified rA3G mutants showing these mutants displayed reduced deaminase activity at various levels. This reduction of deaminase activity is also consistent with the similar mutants in hA3G (e.g. rM9 & rM10 of rA3G are similar to to M9 & M10 of hA3G) as assayed in HEK293T cell lysates (original data in Fig. 4E, S-Table 5).

2) We repeated the ssDNA as well as RNA binding assay using EMSA (see updated Kd values in S-Table 3), Overall, the reduction of binding is more severe on ssDNA binding than RNA binding. The lowered ssDNA binding of these mutants of rA3G could account, at least partially, for the various level of disruption of deaminase activity. However, the degree of lowered ssDNA binding does not appear to have a strict correlation with the disruption level of deaminase activity.

3) We also added a new data panel in S-Fig. 10A to show the sensitivity of the hA3G mutants to Vif-mediated degradation. One caveat with this result is that the detectible protein of M9 and M12-M14 constructs appeared to be at low steady state levels even if with increasing amount of transfected DNA after multiple tests with side-side comparison with WT. Based on the observation that M10-M14 showed partial HIV restriction in the absence of Vif but no restriction in the presence of Vif (Fig. 4E), it can be concluded that these mutants should be sensitive to Vif-mediated degradation, and can be packaged into virion to sufficient level to display HIV restriction.

4) While dimerization mutants and PEP for RNA binding mutants may impact HIV restriction activity mainly through affecting the deaminase activity and retain HIV restriction in deaminase-independent manner, dimerization and RNA-binding dependent dimerization/multimerization of A3G is shown to be critical for internal retroelement inhibition (SINEs and LINEs) by several groups. Therefore, the biological relevance of dimerization (and subsequent multimerization) may be more critical for restricting internal retroelements than for external HIV-1.

We have discussed these points with the new supplement data mentioned above in this revision.

Major concerns:

1. Huthoff et al. (PLoS Pathog. 2009) have previously proposed a model of human A3G dimer formed by interaction of two N-terminal domains. Although Huthoff's structural model of hA3G dimer was significantly different from the structure of rA3G dimer, the hA3G dimer model showed some similarities as Y124 and W127 formed an inter-molecular hydrophobic core, and R24 was a part of positively charged inter-molecular interface. Huthoff et al. mutated R24 of hA3G, and observed substantial reduction of both virion encapsidation and HIV-1 restriction (Fig. 6 of Huthoff et al.). These results are substantially different from the results shown in Fig. 4 of this manuscript since disruption of the RNA-binding region containing R24 of hA3G was not critical for virion encapsidation nor HIV-1 restriction. It is appropriate to refer the paper by Huthoff et al , and address those controversial results.

RESPONSE: In the A3G model by Huthoff et al 2009, even though proposed based on the elongated APOBEC2 tetramer structure, W127 and R24 are also located around the interface of the elongated dimer, and mutation of W127 and R24 disrupted dimerization and RNA binding and HIV restriction, however W127 mutation showed a near complete abolishment but R24 mutation has partial effect.

The tetramer structure of the single domain APOBEC2 (A2), in which two dimers of A2 interact with each other mainly through h1-loop1 (vicinity of equivalent of R24 in A3G) and loop7 (vicinity of W127 in A3G) (the red circle in Rp-Fig.1A). There are two fundamental differences between this A2-based A3G dimer model with the crystal A3G dimer structure (see Rp-Fig.1 below). First is the CD1-CD2 arrangement differs dramatically between the A3G model based on the A2 dimer and the A3G structure (Rp-Fig.1A, 1B), which will impact the way two A3G molecule come together (Rp-Fig. 1C, 1D). Second is the local structures and interface interactions of the modeled A3G dimer as shown in Rp-Fig. 1D (and shown in Fig. 4c in Huthoff et al 2009) are different from those observed in the dimer structure of the full-length rA3G. Despite these obvious differences, the general conclusion that R24 and W127 are near the dimer interface is correct.

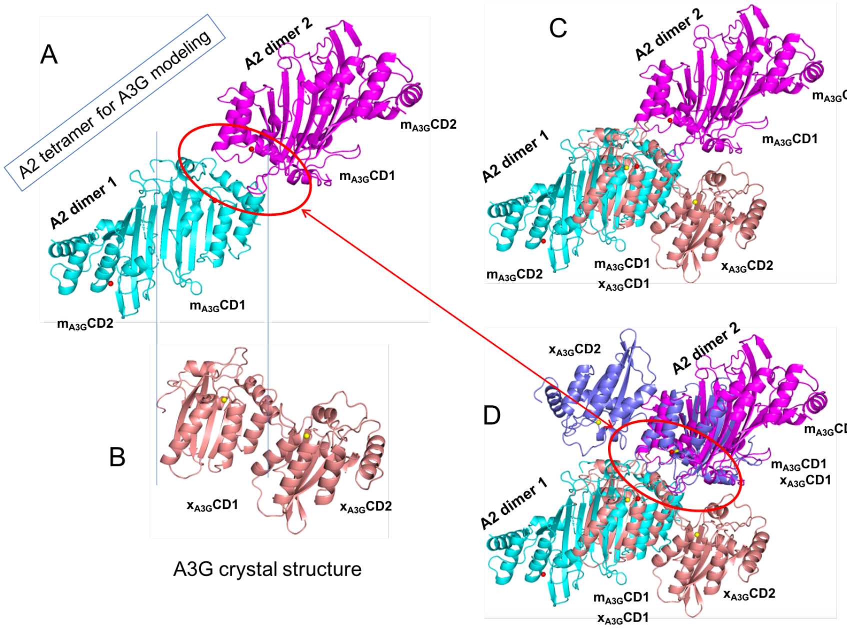

Rp-Fig.1. Comparing A2-based A3G model and the A3G crystal structure. **(A)** The A2 tetramer structure used for A3G modeling. The A2 dimer 1 (or 2) in the tetramer is used for the modeled A3G CD1 (m_{A3G}CD1) and CD2 (m_{A3G}CD2) as one full length A3G molecule. **(B)** A rA3G crystal structures, with its CD1 (x_{A3G}CD1) aligned with the m_{A3G}CD1 on panel-A to show that the crystal structure A3G CD2 (x_{A3G}CD2) is on the right side of CD1 when compared with the left-side positioned m_{A3G}CD1 in the A3G modeled on A2 dimer 1. **(C)** Superimposition of rA3G crystal structure on to the A1 dimer 1 modeled A3G based on x_{A3G}CD1 and modeled m_{A3G}CD1, showing the different position of their corresponding CD2s (x_{A3G}CD2 and m_{A3G}CD2). **(D)** On the basis of panel C, superimposition of another r3G crystal structure on to A2 dimer 2 based on CD1. The modeled A3G dimer face (or the A2 dimer-dimer interface) is indicated by a red circle. Loop 1 (vicinity of R24) and loop 7 (vicinity of W127) of two m_{A3G}CD1s are located at the interface, but their local structures and interface interactions in the model shown here are totally different from those observed in the crystal structure of the full-length rA3G dimer reported in this paper.

Besides the report in Huthoff et al 2009, Pollpeter et al. 2018 from the same group (Nat Microbiol. 2018 Feb;3(2):220-233) further showed that R24A did not show significant difference from WT in HIV restriction activity at higher R14A expression level (Fig. 6b), and its virion packaging can be compensated by raising the A3G expression level. In addition, other groups (Lavens et al 2010, ref 30, Koyama et al 2013 ref 38) all showed R24 mutation had some effect on virion packaging and HIV restriction, even though it's not as critical as W127A.

When comparing our mutants with prior studies on R24A, one difference is our A3G mutants have R24T or R24E in background of other additional mutations; whereas the Huthoff 2009 had single R24A mutation. Thus not entirely comparable. Despite this, R24A restriction activity from Huthoff 2009 was ~50% and ours was ~40%, slightly less but not drastically different.

We added new data and statements in the revision to address these concerns and clarify that these reports on R24 mutant effects are not contradictory to ours in terms of HIV restriction (Fig. 4) as well as out *in vitro* biochemistry results (Fig. 3E and S-Fig. 6). The mutants containing R24 mutations in Figure 4 are M12-14. Due to their low level of detected protein level (even with much more transfected plasmid DNA), it is difficult to determine at what level the packaging is affected. However, it's clear that their HIV restriction activity was reduced compared to WT. Our new data showed that, after lowering the WT expression to the comparable level of M12-14 expression, the WT has about 4-fold higher HIV restriction activity than M12-14 (see data in S-Fig. 10B). In addition, we added description stating that our *in vitro* biochemistry using purified rA3G R24T alone mutant protein showed that R24T produced a substantial more protein in the D (dimer) and M (monomer) fractions than WT, and less protein near the large aggregated V (void volume) RNA-binding fractions than WT before RNase A treatment (S-Fig. 6A, compare SDS-PAGE of the SEC elution fractions for R24T vs WT) – suggesting R24 has certain contribution to RNA binding.

2. Structure of the rA3G E/Q variant may not be biologically relevant for hA3G because Zn is essential for the catalytic reaction, and HIV-1 restriction by hA3G is predominantly achieved through deamination of cytidines in viral DNA.

RESPONSE: Yes, we agree that from deaminase catalytic point of view, the E/Q structure represents catalytically inactive form because it lacks Zn-coordination in its CD2. The lack of Zn-coordination is also observed in two A3F-CD2 wt structures (PDBid: 5HX4 and 5W2M), suggesting that the Zn coordination in A3F-CD2 and A3G-CD2 appear to be less tight (and thus can get lost under certain conditions) than in their CD1 domain and other APOBEC proteins.

We added new data (S-Fig. 3) to investigate if E/Q variant has defect for catalysis activity. The new data show that reverting the E259Q back to wt E259 (E/Q*) enables the variant to be fully active in deamination in HEK293T cell expression lysate (see new data in S-Fig. 3A-D). Additionally, E/Q* plus F126Y, or plus K180S/L184S or plus CD2Δloop3 (i.e. deleting CD2 loop 3) are all as active as E/Q* and WT. These results indicate that the E/Q construct per se is fully active when E259Q is reverted back to E259, and that the crystallized E/Q structure lacking Zn in the CD2 domain should represent a catalytically inactive A3G structural state that may be a crystallographic artifact, or may reflect a naturally existing structural state with a particular function that does not require deaminase activity. We have discussed the newly added data and the possible explanation for the E/Q structure in this revision.

3. In the rA3G FKL structure, the N-terminal and C-terminal domains interact through h3, h4 and loop7 of the N-terminal domain and h1, h2 and β2 and loop3 of the C-terminal/catalytic domain. Since h1, h2 and β2 and loop3 are involved to form the catalytic pocket, the FKL rA3G in the provided structure is likely to have reduced catalytically activity. Does the rA3G FKL structure represent a catalytically active intra-molecular domain orientation?

RESPONSE: Yes, FKL with E259 indeed has the reduced catalytically activity and we added this new data in the revision (S-Fig. 3). We generated the active version of them by mutating back the catalytic E259 residue (called FKL* and E/Q*), and tested the deaminase activity in HEK293T cell lysates (see new data in S-Fig. 3). The results indicated that FKL* is less active than E/Q* and WT. It is possible that the mutations could alter some of the structural features (including CD1-CD2 interaction), and this possibility is stated in this revision. We also added new data (S-Fig.3) to assess the effect of R8, K128D, deletion of CD2loop3, F126Y, K180S/L184S on deaminase activity: (**1**) E/Q* carrying R8 and K128D displayed close to wild-type equivalent

deaminase activity. (**2**) E/Q* carrying individual F126Y mutation, or K180S/L184S, or CD2loop3 deletion, also displayed close to wild-type equivalent deaminase activity. (**3**) Adding CD2loop3 back to the FKL* construct (FKL*+CD2lp3, S-Fig. 3D) showed a slightly enhanced activity but still lower than E/Q*, indicating CD2 loop3 is likely not critically important for the deamination activity in these assay conditions. The lowered activity is likely due to the KL mutation at the dimer-interface, which is consistent with the lowered deaminase activity observed for other dimer-interface mutants of rA3G as shown in S-Fig. 8.

4. The dimer interface found in the full-length rA3G has been already reported by the same laboratory as they have published crystal structure of the N-terminal domain of rA3G (Xiao et al., Nat Commun. 2016). The Xiao et al. paper also discussed the nucleic acid binding at the dimer interface. Authors should emphasize what is new in the full-length rA3G structure.

RESPONSE: When the dimeric structure of rA3G N-terminal domain CD1 alone is determined, it is not clear to us if a full-length rA3G containing CD2 linked to CD1 will contribute to dimerization interaction. With anticipation of additional interactions from CD2 in the full-length construct, we did not analyze the CD1 alone dimer interface thoroughly. When the full-length rA3G shows the same dimer interface as CD1 alone, we performed detailed analysis to reveal four new things: (1) the dramatic enhancement of the positive electrostatic potentials if two monomers dimerize to position R24 and nearby charged residues in close proximity, a phenomenon well characterized for DNA minor groove width change to bring the two phosphate backbone closer that is accompanied with intensified negative electrostatic potentials for increased binding to positively charged protein residues (Rohs et al, Nature, 2009); (2). RNA binding to this area is important for the stabilization of full-length dimer formation; (3) the dimerization mutants unexpectedly can still be encapsidated to show partial HIV restriction; (4) surprisingly, all the dimerization mutants or the PEP mutant that indirectly affected dimerization reduced deaminase activity, as shown in both cell lysate deaminase assays and in proviral mutation rates (Fig. 4E, S-Table 5), as well as in vitro assays using purified equivalent mutants in rA3G (see new data in S-Fig. 8). We tried to state this more clearly in the revised manuscript.

5. rA3G dimer is more stable in solution (Xiao et al., Nat Commun. 2016 and this manuscript) than hA3G dimer since hA3G is most likely under equilibrium of various multimers even after RNAse treatment and size-exclusion column separation (e.g. Iwatani et al., J Virol, 2006; Chelico et a., Nat Stuc. Mol. Biol, 2006; Morse et al., Nat. Commun. 2017). Therefore, oligomeric states of rA3G and hA3G may be different in solution. Extrapolation of dimeric structure of rA3G to hA3G should be considered more carefully since it may or may not be true in hA3G.

RESPONSE: We agree with this insightful comment about the potential difference between rA3G and hA3G in the stability of the dimeric and oligomeric forms. The solubility of rA3G and hA3G in E. coli cells also are obviously different. We have been able to purify soluble hA3G and some of its mutants from insect cells, which does not behave well and displayed polydispersity at concentration around 0.5-1mg/ml or above and did not give us crystals at low or high concentrations. Unfortunately, hA3G behaved even worse in E. coli cells, and we could not purify homogeneous monomeric or dimeric protein from any hA3G wt or mutant constructs from E. coli expression system. That's part of the reason we started the solubility screening of other primate A3Gs, and found that rA3G is more soluble than hA3G in E. coli. The poor solubility of hA3G prevents us to perform the direct analysis of dimerization and RNA binding of hA3G protein in the same way for rA3G. As a result, we resort to rA3G structure as a surrogate to guide our mutational studies of hA3G in cells. At this point, the results of the dimerization interface mutants for rA3G and hA3G all had reduced deaminase activity, which may suggest certain correlation of the similarity of the two proteins and behavior. But in general, we agree with this reviewer on this point, and we added caveat about the potential difference in dimerization and multimerization between rA3G and hA3G versions in this revision.

Reviewer #2 (Remarks to the Author):

This manuscript reported a crystal structure of full-length rhesus macaque A3G. The structure revealed a detailed arrangement of CD1 and CD2, the structural basis for A3G RNA binding and dimer formation. Based on mutagenesis studies, the authors argued that the disruption of the PEP surface in the dimeric interface disrupted RNA binding and A3G dimerization, but had no interference on A3G encapsidation and HIV restriction. Solving the full-length A3G structure is a breakthrough in the A3G research field and will have many important impacts in the area of A3G biology research. While this manuscript is leading in solving the full-length structure, the reviewer has following questions/concerns that should draw the authors' attention.

1. In order to solubilize and crystallize rA3G, several mutations were introduced into the rA3G protein. The eight amino acids, CQKRDGPH, were replaced by four amino acids, AEAE, in the h4 and lp8 region of CD1 (S-Fig. 1). The crystal structures showed that this region was directly involved in the interaction between CD1 and CD2. The E140 interacted with amino acid(s) in CD2 (S-Fig. 3). It raises the reviewer's concern that the mutation might alter the interaction between CD1 and CD2. The authors should address the concern in the discussion.

RESPONSE: We thank the reviewer for the positive and fair reviews and constructive critics. We agree with the reviewer's concern about the possibility of structural alteration by mutations. To help readers understand where were the mutations on the structure, we added S-Fig. 1B with the crystal structure of FKL to show clearly where all the mutations are located on the structure. It is possible that these mutations could alter some of the structural features (including CD1-CD2 interaction), and this possibility is stated in this revision. In addition, we also analyzed the deaminase activity of the E/Q (at E259) and FKL construct by putting back the catalytic E259 (the E259 active form called E/Q* and FKL*), and also by adding more mutated residues to the E/Q* construct to reach all the mutations in the FKL* construct as shown in S-Fig. 1B to assess the effect of these mutations on deaminase activity. The results are shown in S-Fig. 3, which demonstrate that E/Q* has deaminase activity comparable to WT, whereas FKL* has reduce activity possibly due to the dimer-interface mutation, which is also discussed in the Response to point 3 of reviewer 1 above. We have added discussion on this point in the revision.

2. The mutations introduced in hA3G was based on the assumption that rA3G and hA3G have a similar crystal structure. It is plausible to accept this assumption given the high homologous amino acid sequences between them. However, there are some different amino acid residues involved in the interface between rA3G and hA3G as highlighted in red(S-Table 3 and S-Table 4). Therefore, the reviewer thinks it is essential for the authors to provide experimental evidence to confirm the mutants' dimer formation or the RNA bindings as they claimed in the manuscript.

RESPONSE: We agree with the reviewer that because of some of the amino acid differences between the two closely homologous rA3G and hA3G protein, their structure as well as biochemical properties are expected to be similar but not identical. The biochemical property difference is evident in that so far we haven't been able to obtain well behaved hA3G purified from E. coli or insect cell expression for crystallization study. That has led us to search for other primate homologs, which yielded the rA3G structure. As a result, while we were able to characterize the dimer-interface mutants and PEP RNA-binding mutants using purified rA3G, it is still difficult to characterize such equivalent mutants of hA3G due to poor solubility problem. In order to address the question raised by this reviewer, we attempted to get around this solubility problem by generating a rA3G-hA3G chimera: using rA3G as the framework but replace the dimer-interface residues to all of those aligned to hA3G. The alignment reveals that, the only amino acid differences between rA3G and hA3G at the dimer-interface are on helix 6:

```
rA3G: 181-HYTLLQAT-188
hA3G: 181-YYILLHIM-188
```

Therefore, the rA3G helix 6 is replaced with hA3G CD1 helix 6 to generate the rA3G-hA3G$_{h6}$ chimera (H6) that has the exact hA3G residues at the protein-protein dimer interface if hA3G also dimerizes in the same way as rA3G. We then ask if this H6 chimera carrying the all dimer-interface restudies from hA3G behaved similar as

rA3G WT during purification in terms of RNA association and dimer/multimerization with or without RNase A treatment. In the new data shown in S-Fig. 7, this H6 chimera mutant showed similar RNA association to rA3G WT (especially after relatively heavy RNase A treatment, S-Fig. 7A), it also displayed similar dimer/multimerization as rA3G WT before or after RNase A treatment (S-Fig. 7C, 7D). It is worth noting that, while H6 chimera protein also shifted to dimer (D) and monomer (M) fractions after RNase A treatment (SDS gel in S-Fig. 7D), the SEC profile shows more heterogeneous peaks that that of rA3G WT, possibly because hA3G have three interface residues (Y181, I183, I187) that are much more hydrophobic than those in rA3G (H181, T183, A187), and thus less stable/soluble when exposed as monomers. While this data may not be the ideal one to prove that hA3G dimer-interface mutants and PEP area mutants may have the same extend of disruption in RNA binding and dimer/multimer formation as in rA3G mutants, this result suggests that the dimer-interface can be interchangeable between rA3G and hA3G, which leads to the presence of the same PEP area that has essentially the same set of surface residues on both proteins. We have discussed this new data in the revision.

3. On page 10, the authors claimed that "….. the residues mutated in M12-14 to disrupt RNA binding in the PEP and nearby areas on CD1 are not critical for HIV-1 restriction and virion encapsidation,……". What the reviewer saw was that the M12-M14 all had dramatic packaging defect comparing to WT and M1 in Fig 4A. The authors should repeat this experiment with a similar expression level of all A3G proteins. If it is not possible to enhance M12-14 expression, what about reducing other protein expression? Again, the binding of M12-M14 with RNA needs to be confirmed by experiment.

RESPONSE: About the expression level, indeed after several attempts, it appeared that it's not possible to enhance M12-M14 expression level for reason not well understood. We then tried to lower the WT hA3G expression level to approximate those of M12-M14 (see new data in S-Fig. 10B). This resulted in similar levels of WT A3G being encapsidated in the virion as M11-M14. At this condition, we observed approximately 4-fold more HIV-1 restriction by WT. These data suggest that the mutants are unable to restrict HIV-1 as well as WT A3G at the similar protein expression level, which is consistent with their lower deamination activity and deamination-independent activity. With M12-M14 behavior, because it's not possible to obtain well-behaved proteins of hA3G and its mutants, we generated equivalent mutants rM13, rM14 (rM12 was tested before) of rA3G, and characterized their RNA association and dimer/multimerization with and without RNase A treatment during purification (see new data in S-Fig. 7). The new data show that in rM13 and rM14 RNA binding and multimerization are impaired (S-Fig. 7). We have discussed this new data in the revision.

4. minor issues:

Western blot of cell lysates and virion should be labeled in Fig 4 A and B
On page 9, 3rd line in the last paragraph, "M2 and M4 both had significantly lower HIV-1…." should be "M2 and M3 both….."? Reference 44 has been retracted (i.e. Chiu et al., Nature, 2005).

RESPONSE: We have re-labeled Fig. 4A, 4B. Also M2 and M3… on page 9 has been corrected. Thank you for pointing these out.

With regard to the citation of the retracted reference (Chiu et al., Nature, 2005), our understanding of the retraction is because of the wrong conclusion on "APOBEC3G functions as a post-entry restriction factor in resting CD4 T cells". Their other data on the HMM and LMM conversion after RNase A is correct, and has been repeated by others (including us). That's why we cited the paper (and two other papers) for the HMM/LMM statement in our original version even if the paper was retracted. However, given this reviewer comment, we have deleted this cited reference.

Reviewer #3 (Remarks to the Author):

In the manuscript, Yang et al. present their work on determining the crystal structure of a full-length two-domain A3 protein, Macaca mulatta A3G. They find that the two domains are flexible around a five amino acid linker, with one conformation contorts the fold of the catalytically active domain. They show that a positive electrostatic patch is formed at the dimer interface and is involved in RNA binding, and disrupting RNA binding and oligomerization can be separated from packaging into HIV virions and restriction of HIV. This work is an important advancement for the field of A3 structural studies, as well as HIV research. However, the manuscript would be strengthened if the authors addressed the following concerns:

• Substantial impact in presentation is lost due to the design of the figures. The authors mentioned that they had screened A3G homologs from different primate species and found two engineered rA3G FL constructs with good behavior for structural studies. These mutations should be clearly marked in the structural figures. It would also be very helpful to include a description of the rationales for the design of those mutants, especially the FKL and E/Q mutants which gave the crystal structures, so readers can understand how these crystal structures may vary from the WT rA3G protein.

RESPONSE: We are grateful for the positive and fair reviews and constructive critics from this reviewer. To help clarify what are mutated on the two structures, we added S-Fig. 1B with the crystal structure of FKL to show where the mutations are located on the structure, with a list of mutated residues on both FKL and E/Q constructs. Because the E/Q construct contains the subset of mutations within the FKL construct, the location of mutated residues on E/Q structure can be located using the FKL structure in S-Fig. 1B. A description of how we arrived at the mutations to get the crystals has been added in this revision.

• In Figure 1A & B, it is important to highlight the short liner region connecting CD1 and CD2. In Figure 1D, label h1, β2, loop 3 and 7 which are involved in the CD1-CD2 interaction; Similarly, in Figure 1E, label h6, β2, loop4 and 10.

RESPONSE: We have highlighted the linker in Fig. 1A, 1B, and added the labels to Fig. 1D and 1E.

• Are the differential packing orientations between E/Q and FKL mutants caused by the intrinsic plasticity of the FL rA3G or affected by the mutations introduced (which may lead to different crystal packing interactions)?

RESPONSE: This is an excellent question and we don't have a clear answer to this for now. The differential packing orientations could come from either intrinsic plasticity or the mutation or both. For this reason, mapping the location of the mutated residues on to the structure in S-Fig. 1B may help to present the potential possibilities for the readers. We have also added caveat statement regarding these two possibilities in this revision.

• At the end of the first results section, the authors mentioned that the E/Q CD2 has a much closer packing interaction with CD1, but the buried surface areas are quite similar (623 vs. 700). It would be helpful to elaborate this more.

RESPONSE: Normally, it is expected to have much larger buried interface areas if two interaction domains have a much closer packing interaction. However, in the case of E/Q CD2 packing with CD1, the closer packing (i.e. a center of mass of E/Q CD2 moved closer to CD1 than the FKL CD2, see Fig. below) caused the restructuring of h2 have a much shorter h2 in E/Q structure, as well as the disorder of most part of h2 and loop 3 at the top around Zn-center, leading to the loss of buried surface area, resulting in only slightly increased buried interface area in E/Q. This restructuring of h2 is likely to accommodate the closer packing of CD2 with CD1 as the canonically long h2 would have serious clash with CD1 without changing its conformation. We have elaborated this point in the revision.

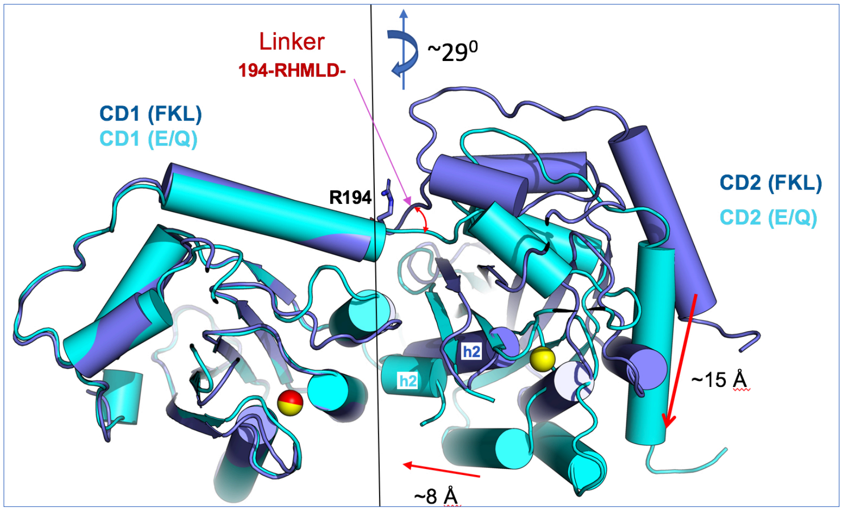

Rp-Fig.2 (also shown as S-Fig. 2A). Superimposition of the FKL (blue) and E/Q (cyan) structures of rA3G. The superimposition is based on CD1 domains to reveal the rotation and angel differences between CD1 and CD2 of the two structures.

• The E/Q structure seems less than likely to represent the deamination competent state since it has kicked out the zinc in its catalytic pocket, does this mean that the conformation of the FKL structure is more biologically relevant? Some discussions would benefit the readers.

RESPONSE: We agree that, because Zn is required for catalysis, the E/Q structure per se represents catalytically inactive form. It is unclear to us if this catalytically inactive structural form has certain functions, such as the non-deaminase related function in restricting retroelement and HIV-1. To test if E/Q variant has structural defects for catalysis or not, we added new data to show that reverting the E/Q back to wt E259 (E/Q*) make the variant fully active in deamination in 293T cell lysate (see new data S-Fig. 3A-D). In addition, E/Q* plus F126Y, or plus K180S/L184S or plus CD2Δloop3 (the original E/Q* construct contains wt CD2 loop3) are all as active as E/Q* and WT. These results suggest that, even though the crystallized form of E/Q represents a catalytically inactive form because of lack of Zn, it appears to be convertible in solution to a Zn-coordinated active form for catalysis as it's full active with a wt E259 residue.

The loss of Zn coordination in E/Q structural form likely is the result of tighter CD2 packing with CD1 which leads to the refolding of h2 and destabilization of loop3 and the associated H257 required for coordinating Zn. Loss of Zn-coordination is also observed in the wild type A3F-CD2 (5HX4, 5W2M), thus the Zn in A3F-CD2 and A3G-CD2 appear to be more loosely coordinated (and thus displaying gain/loss of Zn in the structure) than in their CD1 domains and in other APOBEC proteins.

• It would be interesting to expand more on Figure 3B, rM11? It seems surprising that it still bound RNA when the other interface mutants that only had one different mutation did not bind any RNA at all.

RESPONSE: We think the reviewer may be referring to the comparison of rM11 with rM10 and rM15, all of which are dimer interface mutants, and they have one residue more or less mutated on top of each other, and they showed different phenotype for RNA binding and the associated oligomerization in Fig. 3B. Clearly the extra residue mutation in rM15 (I26A) that directly form hydrophobic packing at protein-protein dimer interface plays an important role in further destabilizing the dimerization and RNA binding, providing a possible explanation for a much reduced RNA binding and loss of most multimerization (also S-Fig. 6A). However, it's harder to compare rM11 with rM10, as the same residues L184-A187 are mutated to DY or SE in rM10 or rM11, respectively. Because rM11 has one more residue mutation (K180S) than rM10 but appears to be less effective in disrupting RNA binding and oligomerization, the interpretation we can think of is that the L184D-A187Y mutation in rM10 is more disruptive than the L184S-A187E in rM11.

However, if examining the detailed oligomerization distribution in S-Fig. 5A (which is the expanded version of Fig. 3B), while rM15 and rM10 showed mostly near the monomeric protein peak (M) position, rM11 also has a significant peak near the monomeric (M) position. The similarity of rM11 to rM10 and rM15 is even more evident when the SDS-PAGE gels of A3G protein distributed in the monomeric peak fractions vs the large RNA-binding oligomeric peak in S-Fig. 5A, majority of the A3G protein is distributed into the M peak fractions for all three constructs, which is different from the WT in which the A3G is mostly distributed in the large aggregated void volume (V) peak. We have added discussion on this point in the revised manuscript.

• In figure 4, was the low expression of M11-M14 taken into account in the infectivity assays? Would those mutants restricted HIV infection to the same level as WT if they had been expressed or packaged at the same amount?

RESPONSE: The plasmid transfection amounts for M11-M14 were 2.5-fold higher than the WT (250 ng vs. 100 ng expression plasmid) to account for lower expression. But their protein levels still could not reach WT levels. We then decreased the amount of plasmid transfected for the WT (25-50 ng of WT plasmid) to lower WT expression level to a similar expression level of M11-M14 (added S-Fig. 10B). This resulted in similar levels of WT A3G being encapsidated in the virion as the M11-M14. At this condition, we observed approximately 4-fold more HIV-1 restriction by the WT. These data suggest that the mutants are unable to restrict HIV-1 as well as WT A3G at the similar protein expression level, which is consistent with their lower deamination activity and deamination-independent activity.

• Figure 4D was first mentioned as a deamination activity result, it does not seem to be correct. Should be Fig 4E?

RESPONSE: Thanks for pointing this out. This has been corrected.

• In figure 4E, the deaminase activity of M1 seems to be omitted. Also, were these deamination levels normalized to the expression level of the protein?

RESPONSE: We did not include M1 for deaminase activity in the cell lysate deaminase assay, with the anticipation that it won't differ much from the WT. As for the comparison of the deaminase activity assay, because of the unexpected change of deaminase activity for the dimer-interface and PEP area mutants, we performed normalization of protein level carefully by first adjusting the expression level of the protein by adjusting the transfected plasmid DNA, and then quantifying the protein level in the cell lysates for further adjustment the lysate amount to be used and normalization for the deaminase assay, and repeated this experiment carefully three times independently.

• There is only one residue in green in S-Fig.1, but the caption says "2 residues in green color are mutated"

RESPONSE: Because the K (K128D) is not in bold, the green is hard to see. We now make the K bold to show the green color better.

• For S-Figure 6 and S-Table 3 – the values generated from the EMSA experiments are **overly precise** with many digits of significant figures and have no errors associated with them. Were these experiments performed in triplicate? More statistical rigor would be needed.

RESPONSE: We agree that the digits used for the Kd value as estimated by EMSA are unnecessary precise. We have repeated the EMSA assay in S-Fig. 6 and provided shorter digits for the estimated Kd value with errors indicated.

• For S-Table 3, some numbers are with too **many digits of significant figures**, such as **cell parameters, I/SigI, and B-factors**. The resolution cutoffs are too conservative: I/SigI from 2.1 to 3.7. Higher resolution data

can be included to improve the precision of the structure. The use of CC1/2 values has justified the inclusion of more high-resolution, weak data (sometimes with I/SigI <1) in refinement (see Karplus et al. Science 2012, doi: 10.1126/science.1218231). Although the quality of the reported structure is acceptable and the potential additional improvement is not expected to change any major conclusions in the manuscript, a more precise model can serve better for those in the community who may use the structural model to facilitate their research.

RESPONSE: We agree with the reviewer, and the digits are overly long for the resolution. The numbers have been changed accordingly (see S-Table 2). Also we agree that the data resolution could be further extended to the resolution bin with I/SigI to around 1.0. Following the reviewer's suggestion, we reprocessed the data. And we realized that the current resolutions are the highest with the reasonable completeness, which used the data to the image edge already. We also tried to process the data and use all the spots to the image corner. But it failed with low completeness. The current resolution of the E/Q and FKL structures reached 2.4Å and 2.47Å, which is sufficiently high for determining the near atomic structure of these protein. In the future, we would like to follow the reviewer's suggestion to collect the highest possible resolution data for future work.

• It would be interesting to see an overlay between the E/Q CD2 domain and other A3 structures – such as PDBIDs 5HX4, 5W3V, 6BUX, and any of the A3G CD2 structures.

RESPONSE: We have performed the alignment of E/Q CD2 with PDBIDs [1](Zn-free A3F-CD2), 5W3V (A3H), 6BUX (A3G-CD2+ssDNA), Zn-bound and Zn-free A3F-CD2 (5W2M) as well as apo-A3G structures 3IR2 and 3IQS. Except E/Q CD2, all other APOBEC domains mentioned here (including the Zn-free A3F-CD2) align with each other very well (S-Fig. 2E, 2F). The Zn-free A3F structure also align almost identically with Zn-bound A3F CD2 (5W2M). The Zn-free E/Q CD2 does not align well in the h2-loops 3, 4 area with apoA3G-CD2 or ssDNA bound A3G-CD2.

REVIEWERS' COMMENTS:

Reviewer #1 (Remarks to the Author):

The reviewer's concerns have been addressed in the point-by-point letter for reviewers.

The revised manuscript is significantly improved from the initial submission by adding new experimental data. Specifically, the authors are to be credited with (1) additional experimental data of in vitro catalytic activity of rA3G and variants (S-Fig.8), and binding constants for ssRNA and ssDNA (S-table 3), (2) additional discussion regarding previously suggested structural model of hA3G dimer, (3) finding that the FKL rA3G variant has reduced deamination efficiency, which may be corelated with intra-molecular interaction between NTD and CTD, and (4) new size-exclusion chromatography data of rA3G-hA3Gh6 chimera variant (S-Fig. 7) which suggests differences in dimerization and multimerization between rA3G and hA3G.

Minor point:
Page 8 line 290, H261 is H216.

Reviewer #2 (Remarks to the Author):

All the concerns have been well addressed. The reviewer recommends the manuscript to be accepted for publication.

Reviewer #3 (Remarks to the Author):

The authors have addressed my previous concerns and the work is now suitable for publication.

Point-by-point response to Reviewer's concern.

Reviewer points:

Only Reviewer 1 raised one Minor point (typo).

- Page 8 line 290, H261 is H216.

RESPONSE: We have taken care of this correction.
